# CD44 Contributes to the Regulation of MDR1 Protein and Doxorubicin Chemoresistance in Osteosarcoma

**DOI:** 10.3390/ijms23158616

**Published:** 2022-08-03

**Authors:** Monserrat Gerardo-Ramírez, Friederike L. Keggenhoff, Vanessa Giam, Diana Becker, Marco Groth, Nils Hartmann, Beate K. Straub, Helen Morrison, Peter R. Galle, Jens U. Marquardt, Peter Herrlich, Monika Hartmann

**Affiliations:** 1 Department of Medicine I, University Medical Center of the Johannes Gutenberg University, 55131 Mainz, Germany; monsemgr@hotmail.com (M.G.-R.); f.keggenhoff@imb-mainz.de (F.L.K.); vanessa.giam@outlook.com (V.G.); beckerdi@uni-mainz.de (D.B.); peter.galle@unimedizin-mainz.de (P.R.G.); 2 Leibniz Institute on Aging-Fritz Lipmann Institute (FLI), 07745 Jena, Germany; marco.groth@leibniz-fli.de (M.G.); helen.morrison@leibniz-fli.de (H.M.); peter.herrlich@leibniz-fli.de (P.H.); 3 Institute of Pathology, University Medical Center of the Johannes Gutenberg University, 55131 Mainz, Germany; nils.hartmann@unimedizin-mainz.de (N.H.); beate.straub@unimedizin-mainz.de (B.K.S.); 4 Faculty of Biological Sciences, Friedrich-Schiller University, 07745 Jena, Germany; 5 Department of Medicine I, University Medical Center Schleswig-Holstein-Campus Lübeck, 23558 Lübeck, Germany; jens.marquardt@uksh.de

**Keywords:** CD44, MDR1, bone tumors, osteosarcoma, hyaluronic acid, proteolytic cleavage

## Abstract

Osteosarcoma is the most common type of pediatric bone tumor. Despite great advances in chemotherapy during the past decades, the survival rates of osteosarcoma patients remain unsatisfactory. Drug resistance is one of the main reasons, leading to treatment failure and poor prognosis. Previous reports correlated expression of cluster of differentiation 44 (CD44) with drug resistance and poor survival of osteosarcoma patients, however the underlying mechanisms are poorly defined. Here, we investigated the role of CD44 in the regulation of drug chemoresistance, using osteosarcoma cells isolated from mice carrying a mutation of the tumor suppressor neurofibromatosis type 2 (*Nf2*) gene. CD44 expression was knocked-down in the cells using CRISPR/Cas9 approach. Subsequently, CD44 isoforms and mutants were re-introduced to investigate CD44-dependent processes. Sensitivity to doxorubicin was analyzed in the osteosarcoma cells with modified CD44 expression by immunoblot, colony formation- and WST-1 assay. To dissect the molecular alterations induced by deletion of *Cd44*, RNA sequencing was performed on *Cd44*-positive and *Cd44*-negative primary osteosarcoma tissues isolated from *Nf2*-mutant mice. Subsequently, expression of candidate genes was evaluated by quantitative reverse transcription PCR (qRT-PCR). Our results indicate that CD44 increases the resistance of osteosarcoma cells to doxorubicin by up-regulating the levels of multidrug resistance (MDR) 1 protein expression, and suggest the role of proteolytically released CD44 intracellular domain, and hyaluronan interactions in this process. Moreover, high throughput sequencing analysis identified differential regulation of several apoptosis-related genes in *Cd44*-positive and -negative primary osteosarcomas, including p53 apoptosis effector related to PMP-22 (*Perp*). Deletion of *Cd44* in osteosarcoma cells led to doxorubicin-dependent p53 activation and a profound increase in *Perp* mRNA expression. Overall, our results suggest that CD44 might be an important regulator of drug resistance and suggest that targeting CD44 can sensitize osteosarcoma to standard chemotherapy.

## 1. Introduction

Osteosarcoma is the most frequent primary malignant bone tumor that predominantly occurs in children and adolescents [1]. Conventional osteosarcoma treatment protocols include neoadjuvant chemotherapy, surgery and adjuvant chemotherapy [2]. Doxorubicin, cisplatin and methotrexate are the most commonly used chemotherapy drugs in the treatment of osteosarcoma [3]. Unfortunately, more than 35% of osteosarcoma patients do not respond to chemotherapy treatments, with a 5-year survival rate at only 5–20% [1,4]. Despite great advances in chemotherapy protocols during the past decades, the survival rates of osteosarcoma patients with recurrent or metastatic disease remain unsatisfactory. Therefore, elucidating mechanisms of resistance is highly desirable in order to sensitize osteosarcoma to chemotherapy and improve patients’ survival.

Cancer cells can achieve therapy resistance by many different mechanisms, depending on the agent and its cellular target. Chemotherapy resistance in osteosarcoma can be linked to mutations in the drug target, alterations in drug metabolism, inhibition of cell death, DNA damage repair mechanisms, changes in signal transduction, the tumor microenvironment or immunity [5]. Frequently, these mechanisms render tumor cells not only resistant to a specific drug, but also to a wide range of structurally unrelated drugs. This is called multidrug resistance (MDR) [5].

Overexpression of members of the ATP-binding cassette (ABC) family of efflux transporters is a common mechanism of multidrug resistance in cancer cells [5]. The ABC family members transport diverse substrates (e.g., ions, amino acids, peptides, lipids, sugars and xenobiotic) across the cellular membranes using ATPase transporter [6]. Four members of ABC family have been shown to contribute to multidrug resistance in osteosarcoma, including MDR1 (also called P-glycoprotein, P-gp or ABCB1), multidrug resistance-associated protein 1 and 2 (MRP1/ABCC1 and MRP2/ABCC2), and breast cancer resistance protein (BCRP/ABCG2) [5]. In particular, high levels of MDR1 were correlated with resistance to doxorubicin [7] and poor relapse-free and overall survival in osteosarcoma [8,9,10].

Expression of cluster of differentiation 44 (CD44) has been implicated in drug resistance, although the direct mechanism of action in osteosarcoma remains unclear [11,12,13,14,15,16,17]. This prompted us to investigate the relative contribution of CD44-dependent pathways to chemoresistance in osteosarcoma cells. CD44 is a group of type I transmembrane glycoproteins encoded by a single gene, which undergoes alternative splicing leading to multiple protein isoforms. The extracellular domain of CD44 serves as a platform for interactions with multiple binding partners, including components of the extracellular matrix, growth factors, matrix metalloproteinases and osteopontin [18]. Insertion of sequences encoded by the alternatively spliced variant exons of CD44, and extensive glycosylation of the ectodomain, contribute to structural and functional diversity of CD44 proteins [19]. The ectodomain of CD44 is followed by the transmembrane region—implicated in protein dimerization—and by a short cytoplasmic domain [20]. The intracellular domain of CD44 (CD44ICD) interacts with proteins such as ankyrin and so-called ERM (ezrin, radixin, moesin) proteins that link CD44 to spectrin and actin cytoskeleton, respectively [18]. The tumor suppressor protein, Merlin (encoded by the neurofibromatosis type 2 gene, *NF2*), which is related to the ERM family, competes with ERM proteins for the same binding site within the cytoplasmic domain of CD44 [21]. The interactions of CD44 with its intracellular binding partners are regulated via phosphorylation, which in turn depends on growth conditions [18]. Specifically, in growth promoting conditions, CD44 forms a complex with ERM proteins and receptor tyrosine kinases, including c-Met, to promote mitogenic signaling, while the interaction of CD44 with Merlin occurs upon growth arrest [21,22]. CD44 co-receptor function is attributed to certain CD44 isoforms containing sequences encoded by variable exons v3 and v6 [18]. In addition, it is probable that all CD44 isoforms undergo sequential proteolytic cleavages, which may influence cellular signaling, independent of receptor tyrosine kinases. In the first step, the ectodomain of CD44 is cleaved off by metalloproteinases. The second intramembranous cleavage by γ-secretase results in the release of CD44ICD for signal transduction [20,23,24,25]. Increased levels of CD44 are associated with invasive properties of tumor cells and predict a poor prognosis in diverse cancer subtypes [26,27,28,29,30,31,32]. There are contradictory reports concerning the function of CD44 in osteosarcoma; however, most studies correlated CD44 expression with drug resistance, recurrence, higher potential of metastasis and poor survival [33,34,35,36]. In particular, expression of CD44 was correlated with resistance of human osteosarcoma cells to doxorubicin treatment, but the underlying mechanisms remain unclear [34,37]. The interaction partner of CD44, Merlin, was also implicated in osteosarcoma. Deletion of the *Nf2* gene, encoding Merlin, leads to the generation of highly metastatic osteosarcoma tumors in mice [38]. The role of *NF2* in the pathogenesis of human osteosarcoma is less well described. *NF2* mutations were identified in single human patients; however, comprehensive studies providing frequencies of *NF2* gene mutations in human osteosarcoma patients are missing [39]. Nevertheless, a broad meta-analysis study demonstrated that *NF2* expression is downregulated in a large panel of human osteosarcomas, which is compatible with its function as a tumor suppressor in this tumor type [40,41]. *NF2* downregulation could be due to either mutations or transcriptional inactivation [40]. Absence of the *Cd44* gene prevents osteosarcoma metastasis in mice with a mutation of the tumor suppressor gene *Tp53* [42] and, as we have shown recently, *Nf2* [38]. In the current study, we applied osteosarcoma tissues and cells isolated from mice with a mutation of the *Nf2* gene, to evaluate the function of CD44 in the resistance to chemotherapy. Our results show that CD44 contributes to multidrug resistance in osteosarcoma by upregulation of MDR1 protein expression, and inhibition of genes belonging to the p53 pathway.

## 2. Results

**Figure 1:**
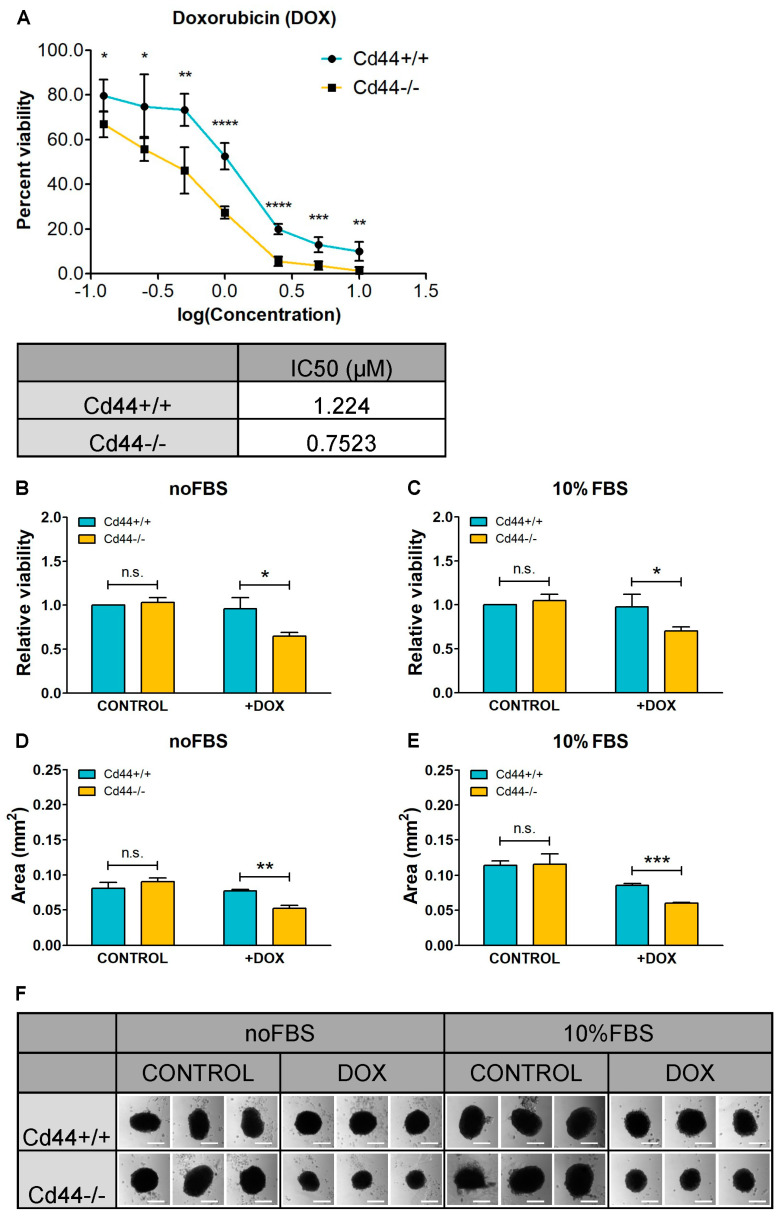
Knockout of *Cd44* sensitizes osteosarcoma cells to doxorubicin (DOX) treatment. (**A**) Determination of the half-maximal (50%) inhibitory concentration (IC50) of DOX. Dose-response curve of *Cd44*-positive and *Cd44*-negative osteosarcoma cells to DOX (upper panel) and corresponding IC50 values (lower panel) are shown. Cell viability was measured in quintuplicates in five independent experiments using WST1 assay. IC50 was calculated using GraphPad Prism and nonlinear regression. (**B**,**F**) Influence of DOX on formation of *Cd44*-positive and *Cd44*-negative spherical colonies. The histograms represent relative mean values of cell viability (**B**,**C**) or surface area (**D**,**E**) ±SD from three independent colony formation assays. Representative pictures of colonies formed in the presence or absence of DOX, either upon serum starvation or in medium supplemented with 10% fetal bovine serum (FBS) are shown in (**F**). Scale bar 200 µm. Student’s *t*-test values: n.s.: *p* > 0.05, *: *p* ≤ 0.05, **: *p* ≤ 0.01, ***: *p* ≤ 0.001, ****: *p* ≤ 0.0001.

In order to study the relevance of CD44 in mediating chemoresistance, we previously isolated osteosarcoma cells from mice that developed endogenous osteosarcoma, due to a mutation of the tumor suppressor gene *Nf2* [38]. To enable the analysis of CD44-dependent processes in genetically comparable tumor backgrounds, *Cd44* was deleted in *Nf2*-deficient osteosarcoma cells, using the CRISPR/Cas9 approach [38]. Our results show that *Cd44*-positive cells were more resistant to doxorubicin treatment than the *Cd44*-negative cells (IC50 of 1.224 versus 0.7523 µM, respectively, *p* = 0.000793) (Figure 1A). We also measured doxorubicin sensitivity in three-dimensional cell culture, which was shown to better mimic the drug sensitivity/resistance behavior of cancer cells found in solid tumors in vivo, than the conventional two-dimensional monolayer conditions (reviewed by Weiswald et al. [1]). Osteosarcoma cells grown under nonadherent conditions formed one single spherical colony per well of a 96-well plate. Doxorubicin-treated *Cd44*-positive osteosarcoma cells formed significantly larger colonies than their *Cd44*-negative counterparts, independent of medium supplementation with serum. The size of *Cd44*-positive and *Cd44*–negative colonies was not significantly different in the absence of doxorubicin (Figure 1B–F).

**Figure 2:**
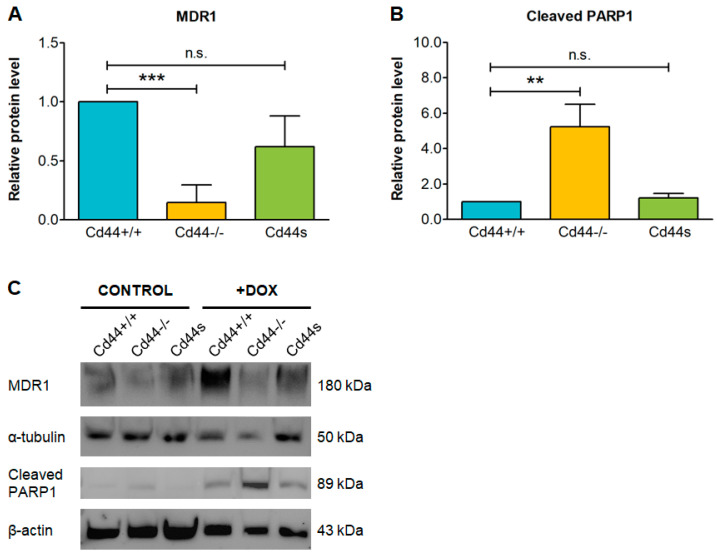
The shortest, standard isoform of CD44 (CD44s) is sufficient to confer resistance to doxorubicin (DOX). Osteosarcoma cells were seeded at 60% confluency in DMEM medium, supplemented with 7% FBS. The cells were incubated for 24 h in the presence of 1 µM DOX or pure solvent (control) and then subjected to immunoblot. The histograms in (**A**,**B**) show mean values of MDR1 or cleaved PAPR1 protein levels ± SD from three independent experiments. The values were normalized to loading control. Student’s *t*-test values: n.s.: *p* > 0.05, **: *p* ≤ 0.01, ***: *p* ≤ 0.001. Representative immunoblot is shown in (**C**).

Moreover, the protein level of MDR1 was significantly reduced in *Cd44*-minus cells, compared to *Cd44*-plus cells, and correlated with higher level of the apoptotic marker cleaved PARP1 (Figure 2). The levels of MDR1 protein could be reconstituted in *Cd44*-negative osteosarcoma cells by re-introduction of the smallest isoform of CD44- the so-called standard isoform (CD44s), suggesting that this isoform was sufficient to confer doxorubicin resistance in osteosarcoma cells (Figure 2A,C). Correspondingly, doxorubicin-treated cells expressing CD44s released low levels of cleaved PARP1, which were comparable to the levels in *Cd44*+/+ cells, expressing wild-type CD44 (Figure 2B,C).

**Figure 3:**
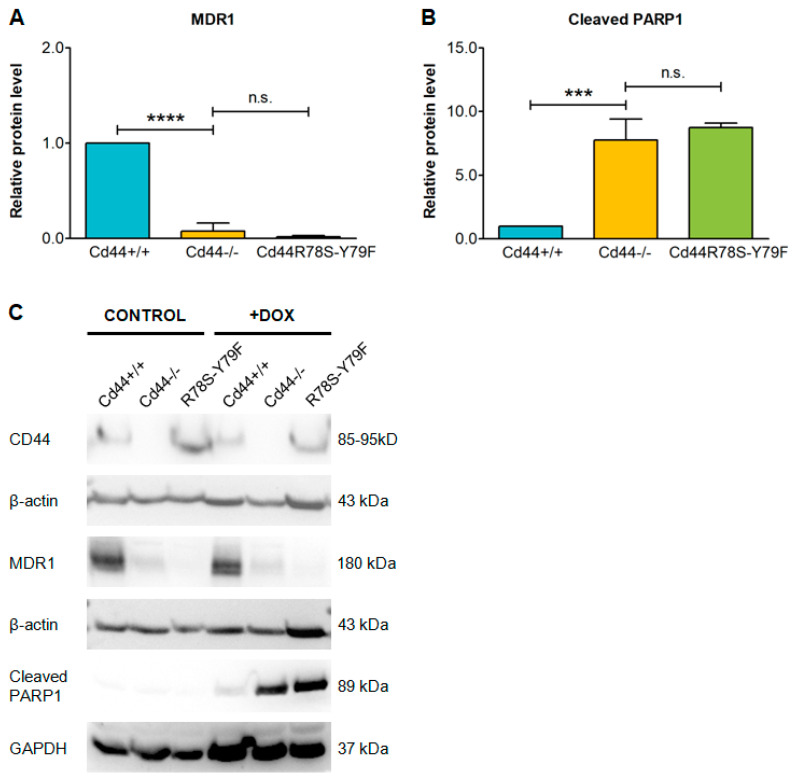
Mutation of the hyaluronic acid (HA) binding site in CD44 ectodomain sensitizes osteosarcoma cells to doxorubicin. *Cd44*-positive wild-type cells, *Cd44*-negative cells, and cells with reconstituted expression of a mutant of CD44 unable to bind HA (Cd44s-R78S-Y79F) were seeded at 60% confluency in DMEM medium supplemented with 7% FBS. The cells were incubated for 24 h with pure solvents (controls) or 1.5 µM DOX and then subjected to immunoblot. The histograms in (**A**,**B**) show mean values of MDR1 or cleaved PAPR1 protein levels ± SD from four independent experiments. The values were normalized to loading control. Student’s *t*-test values: n.s.: *p* > 0.05, ***: *p* ≤ 0.001, ****: *p* ≤ 0.0001. Representative immunoblot is shown in (**C**).

CD44 serves as a major receptor for hyaluronic acid (HA). Binding of CD44 to HA plays an important role in cell adhesion and signaling [43]. To test involvement of the CD44-HA interaction in chemoresistance, we generated a mutant of CD44 that is unable to bind HA [44]. Re-introduction of this HA-binding deficient mutant, CD44s-R78S-Y79F into *Cd44*−/− cells was not sufficient to reconstitute MDR1 protein levels in those cells (Figure 3A,C). Consistently, cells expressing CD44s-R78S-Y79F mutant released similar levels of cleaved PARP1 as the *Cd44*-deficient cells (Figure 3B,C), suggesting that an intact HA-binding site of CD44 is indispensable for conferring doxorubicin chemoresistance.

Our experiments involving cells with manipulated CD44 expression indicate that CD44 may regulate MDR1 function by changing its expression, either on the transcriptional or protein level. The intracellular domain of CD44 (CD44ICD), which is released by proteolytic cleavage, can act as transcriptional co-activator, and could potentially activate MDR1 gene expression [24,45]. In particular, it has been demonstrated that CD44ICD potentiates transcription of genes containing consensus sequence 5′-CCTGCG-3′, known as the CD44ICD response element (CIRE) [45], or pseudo-palindromic DNA sequence 5′-TGAC/GTCA-3′, known as the 12-O-tetradecanoylphorbol-13-acetate response element (TRE) [24,46]. MDR1 protein is encoded by *ABCB1* in humans and *Abcb1a* and *Abcb1b* genes in mice [4].

**Figure 4:**
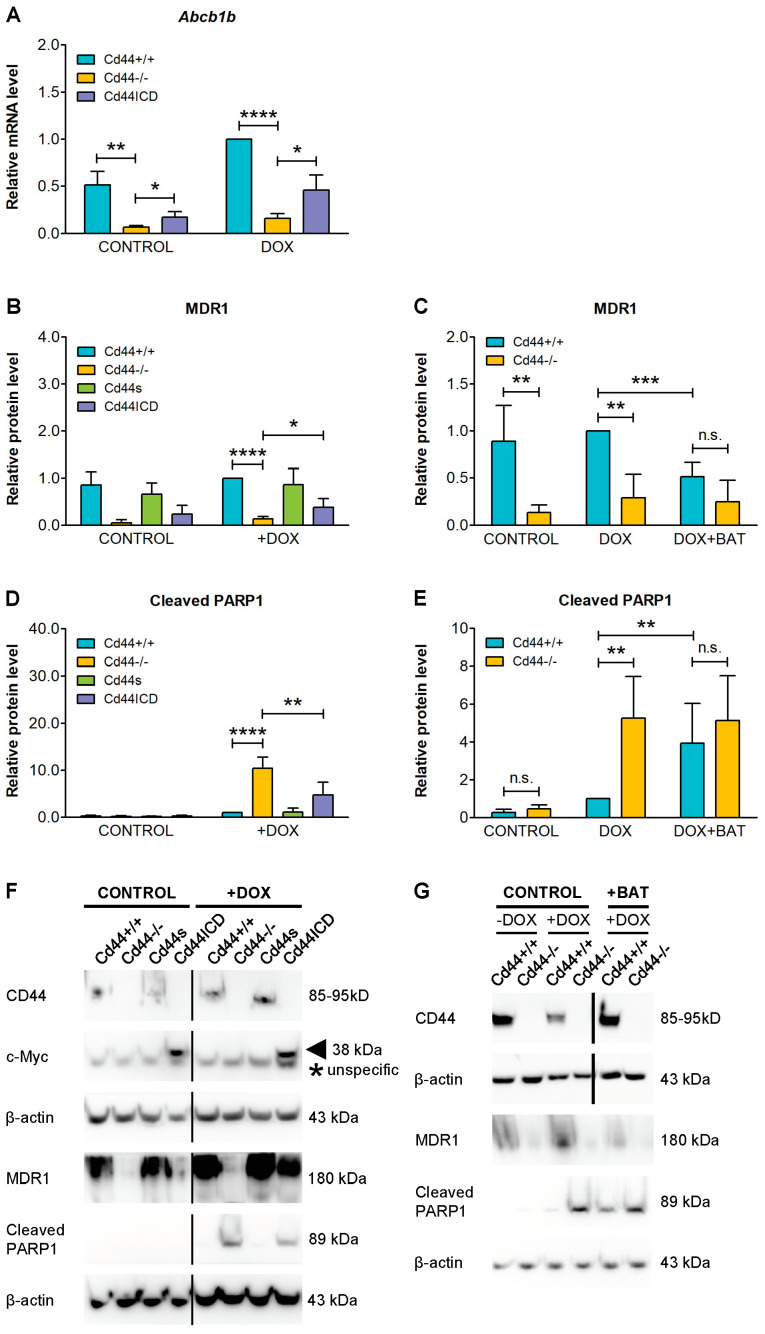
Involvement of CD44 proteolytic cleavage in the regulation of MDR1 expression and doxorubicin (DOX) chemoresistance. (**A**) CD44 potentiates *Abcb1b* gene transcription. (**B**–**G**) Soluble intracellular domain of CD44 (Cd44ICD), mimicking cleavage product, significantly increases resistance to DOX; whereas, treatment with matrix metalloproteinase inhibitor batimastat (BAT) sensitizes *Cd44*-positive osteosarcoma cells to DOX. *Cd44*-positive wild-type cells, *Cd44*-negative cells, and cells with reconstituted expression of Cd44s and Cd44ICD were seeded at 60% confluency in DMEM medium supplemented with 7% FBS. The cells were incubated for 24 h with pure solvents (controls), 1.5 µM DOX or 10 µM batimastat and then harvested for immunoblot or RNA isolation. The histograms in (**A**–**E**) show mean values of mRNA or protein levels ± SD from three (**A**), four (**B**,**C**,**E**) or five (**D**) independent experiments. The values were normalized to loading control. Student’s *t*-test values: n.s.: *p* > 0.05, *: *p* ≤ 0.05, **: *p* ≤ 0.01, ***: *p* ≤ 0.001, ****: *p* ≤ 0.0001. Representative immunoblots are shown in (**F**,**G**). Arrowhead in (**F**) indicates CD44ICD, asterisk indicates unspecific band.

Analysis of the highly similar mouse *Abcb1a* and *Abcb1b* genes revealed presence of the TRE consensus sequences 5′-TGAGTCA-3′ and 5′-TGACTCA-3′ (respectively) in their promoter regions (see Methods), providing structural basis for the regulation by CD44ICD. Moreover, deletion of CD44 in osteosarcoma cells led to significantly reduced *Abcb1b* mRNA levels (Figure 4A). Interestingly, re-introduction into *Cd44*-negative cells of the CD44ICD, mimicking proteolytic cleavage product, could partially reconstitute the expression of *Abcb1b* (Figure 4A). Thus, we hypothesized that CD44ICD released during proteolytic cleavage potentiates *Abcb1b* transcription.

Consistently with the above observations, overexpression of CD44ICD in *Cd44*-negative osteosarcoma cells could partially re-establish the protein levels of MDR1 and significantly reduced the doxorubicin-induced levels of the apoptotic marker cleaved PARP1 (Figure 4B,D,F).

To further test the involvement of CD44 proteolytic cleavage in the regulation of MDR1 expression and doxorubicin chemoresistance, we blocked metalloproteinases using the specific inhibitor batimastat. We previously demonstrated that batimastat prevents CD44 ectodomain cleavage and subsequent release of CD44ICD [20,25]. We compared doxorubicin-treated osteosarcoma cells with or without addition of batimastat (Figure 4C,E,G). Batimastat partially reduced the level of MDR1 in *Cd44*-positive cells but had no effect on the already low level of MDR1 in *Cd44*-negative cells (Figure 4C,G). Inhibition of MDR1 expression in *Cd44*-positive cells was correlated with increased level of the apoptotic marker cleaved PARP1 (Figure 4E,G, compare line 3 and 5). The batimastat-dependent inhibition of MDR1 expression in *Cd44*-positive cells was only partial, suggesting that additional cleavage-independent mechanisms are also involved in the regulation of MDR1. Moreover, these results are compatible with other observations suggesting that CD44ICD potentiates transcription of target genes, but is not sufficient to induce their expression alone [24,45,47,48].

**Figure 5:**
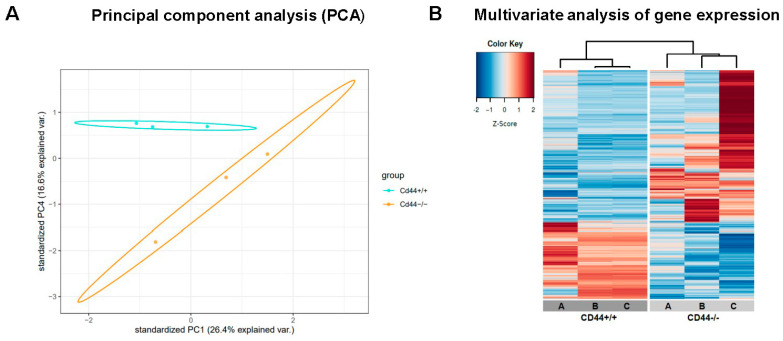

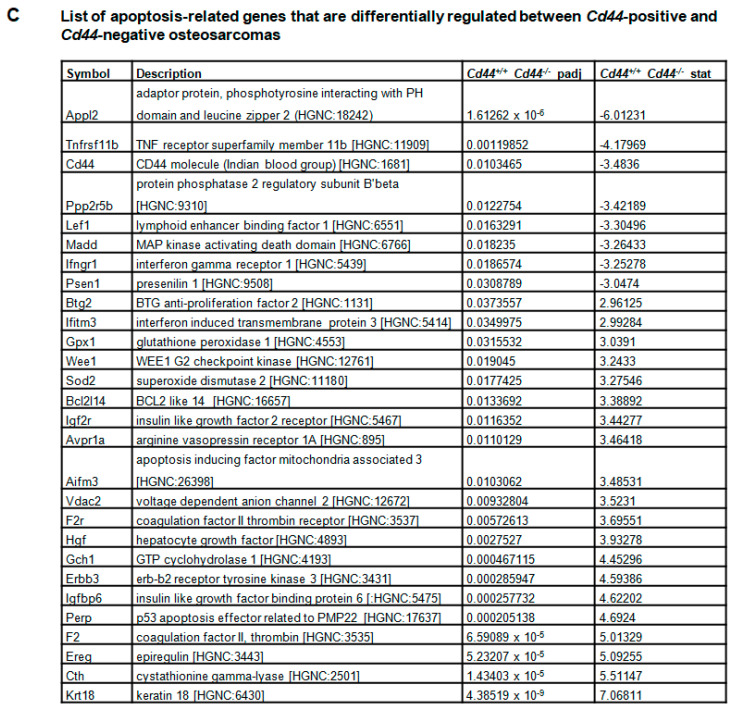
Gene expression analysis. (**A**) Principal component analysis (PCA) of RNA sequencing data. Data were plotted along the first and forth principal components. Each dot in PCA plot indicates a single sample. (**B**) Multivariate analysis of gene expression between *Cd44*-positive and *Cd44*-negative primary osteosarcomas. Unsupervised hierarchical clustering and heatmap showing *Cd44*+/+ and *Cd44*−/− primary osteosarcomas clearly segregated from one another, based on their RNA expression profiles. Three samples of each group were analyzed. Both downregulated (blue) and upregulated (red) RNAs were identified in *Cd44*−/− osteosarcomas. The analysis was performed using an MADE4 package of R software. (**C**) List of apoptosis-related genes that are differentially regulated between *Cd44*-positive and *Cd44*-negative osteosarcomas. The data are represented as Wald-statistic (stat). Genes with negative Wald-statistic values are down-regulated in *Cd44*−/− compared to *Cd44*+/+ primary osteosarcomas, and genes with positive values are upregulated. Benjamin Hochberg adjusted *p*-value (padj) is indicated. Gene description source: HGNC (www.genenames.org/, accessed on 31 May 2022).

Altogether, our results suggest that CD44 contributes to doxorubicin chemoresistance in osteosarcoma cells by regulating MDR1 expression and function. To test if CD44 regulates additional processes that may be involved in chemoresistance regulation, RNA sequencing was performed on *Cd44*-positive and *Cd44*-negative primary osteosarcoma tissues isolated from *Nf2*-mutant mice described previously [38]. Three samples of each group were analyzed. *Cd44*+/+ and *Cd44*−/− primary osteosarcomas clearly segregated from one another based on their RNA expression profiles (Figure 5A,B). A total of 1592 genes were differentially expressed between *Cd44*-positive and *Cd44*-negative osteosarcomas. List of significant genes is included in Table S1.

**Figure 6:**
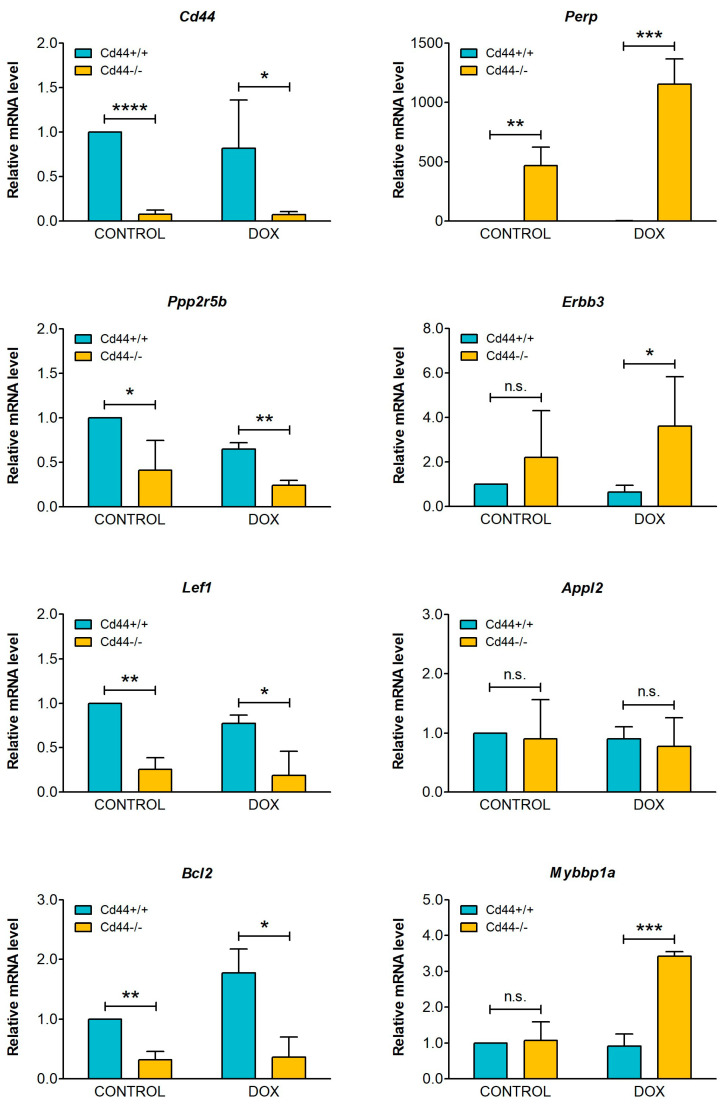
Testing mRNA levels of selected apoptosis-associated genes. *Cd44*-positive wild-type cells, *Cd44*-negative cells, and cells with reconstituted expression of Cd44s and Cd44ICD were seeded at 60% confluency in DMEM medium supplemented with 7% FBS. The cells were incubated for 24 h with pure solvent (controls) or 1.5 µM DOX. The histograms show mean values of mRNA levels ± SD from three independent experiments. Student’s *t*-test values: n.s.: *p* > 0.05, *: *p* ≤ 0.05, **: *p* ≤ 0.01, ***: *p* ≤ 0.001, ****: *p* ≤ 0.0001.

The list of differentially regulated genes contains both those with positive and negative regulatory function in apoptosis (Figure 5C). Expression of selected candidate genes was evaluated by real-time qRT-PCR (Figure 6). The apoptosis conditions were triggered in the osteosarcoma cells using doxorubicin.

One of the top ranked genes upregulated in *Cd44*−/− osteosarcoma tissues was *Perp* (p53 apoptosis effector related to PMP22) (Figure 5C). PERP is a tetraspan plasma membrane protein that belongs to lesser-known transcriptional targets of p53 and p63 [49]. PERP was originally identified as a p53 target specifically trans-activated during apoptosis, but not during cell-cycle arrest [49]. Expression of PERP is downregulated in numerous cancers, suggesting that PERP is a tumor suppressor protein [49]. By real-time qRT-PCR we could confirm that the levels of *Perp* mRNA were highly upregulated in *Cd44*-negative osteosarcoma cells when compared with *Cd44*-positive cells (Figure 6). We also tested expression of *Bcl2*, which is an important anti-apoptotic factor previously correlated with CD44 expression in other human cancers [50,51]. Expression of BCL2 was shown to be highly upregulated in many cancers as compared to normal tissue, suggesting its tumor promoting role [52]. We observed that *Cd44*-positive osteosarcoma cells expressed significantly higher levels of *Bcl2* mRNA, compared to *Cd44*-negative osteosarcoma cells (Figure 6), consistent with the pro-survival function of CD44. However, we could not confirm this effect in the osteosarcoma tissues which were not treated with doxorubicin.

We also investigated the gene encoding nuclear Myb-binding protein 1a (*Mybbp1a*). We found it especially interesting because Mybbp1a binds p53 to activate anoikis in p53-dependent manner [53]. Anoikis is a form of programmed cell death that occurs in anchorage-dependent cells when they lose contact from surrounding extracellular matrix [54]. It is a critical mechanism in preventing dysplastic cell growth or attachment to an inappropriate matrix [54]. Interestingly, the levels of *Mybbp1a* mRNA were significantly lower in doxorubicin-treated *Cd44*-positive cells when compared to *Cd44*-negative cells (Figure 6), indicating that *Cd44*-positive cells might be more resistant to anoikis.

In addition, we confirmed differential expression of *Ppp2r5b*, *Erbb3* and *Lef1* genes in cell culture (Figure 6). Expression of several other genes, including *Appl2* was not significantly different in *Cd44*-positive and *Cd44*-negative osteosarcoma cells (Figure 6), although they were found to be differentially expressed in the tissue (Figure 5). This may implicate that the levels of *Appl2* were influenced by tumor microenvironment, and we could not recapitulate the appropriate conditions in cell culture.

**Figure 7:**
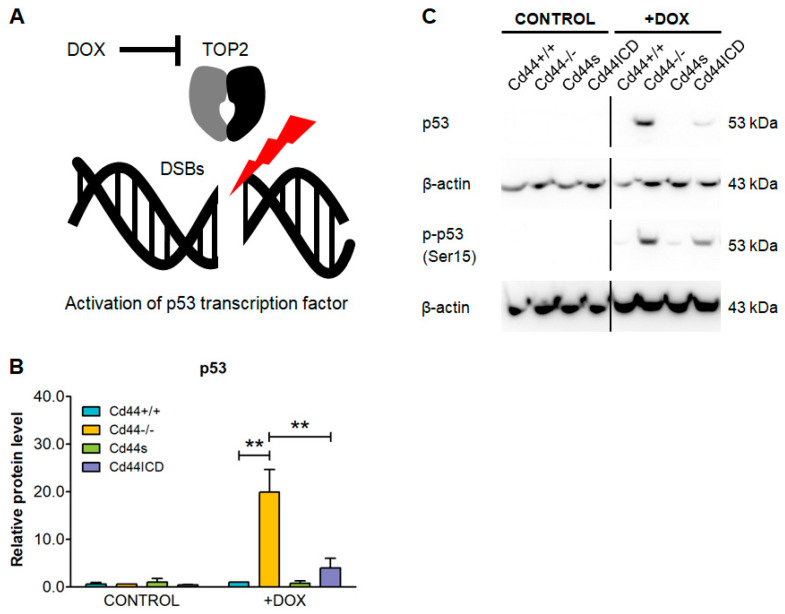
Testing the effect of doxorubicin (DOX) on p53 level in osteosarcoma cells. (**A**) DOX inhibits topoisomerase II (TOP2) leading to DNA damage in the form of double-strand breaks (DSBs). As a consequence, the DNA damage response and p53 pathways are activated, which leads to cell cycle arrest and cell death. (**B**,**C**) DOX increases levels of active p53 in *Cd44*-negative osteosarcoma cells. The cells were treated as in Figure 4. The histogram in (**B**) shows mean value of p53 protein level ±SD from three independent experiments. The values were normalized to loading control. Student’s *t*-test values: **: *p* ≤ 0.01. Representative immunoblots for detection of total and phosphorylated p53 are shown in (**C**). Please notice that phosphorylated p53 was detected by reprobing lower membrane part from the experiment in Figure 4F, therefore the β-actin is the same.

Our results show that at least two of the identified apoptosis-related genes, which were up-regulated in *Cd44*-knockout cells, belong to the p53 pathway. This might be relevant for the regulation of sensitivity to doxorubicin, which acts by inhibiting Topoisomerase II, leading to double-strand DNA breaks and activation of the DNA damage response, involving p53 [2,55] (Figure 7A). To further elucidate the involvement of p53 pathway in doxorubicin-mediated apoptosis, we next asked if the levels of p53 differ between *Cd44*-positive and *Cd44*-negative osteosarcomas. Indeed, expression of p53 was highly induced in *Cd44*-negative osteosarcoma cells (Figure 7B,C), and correlated with high levels of cleaved PARP1 (Figure 4D,F).

Altogether, our studies indicate that CD44 contributes to increased doxorubicin chemoresistance in osteosarcoma, by positive regulation of MDR1 expression and its effects on apoptosis-regulatory proteins, including members of the p53-pathway.

## 3. Discussion

Regulation of MDR1 by CD44 was previously demonstrated in human breast and ovarian tumor cells, as well as in head and neck squamous cell carcinoma (HNSCC) [11,12,13,14,15,56,57]. Table S2 summarizes the molecular pathways implicated in the regulation of MDR1 by CD44. It has been demonstrated that MDR1 and CD44 proteins are physically associated in tumor cells and in yeast two-hybrid system [15,57,58]. Interaction of CD44 with HA initiates a number of signaling pathways that lead to activation of pro-survival signaling and stimulation of MDR1 expression, and function leading to drug resistance [11,12,13,51,56,57]. In this study, we confirmed that CD44 contributes to drug chemoresistance in osteosarcoma cells by upregulating MDR1 levels and influencing genes implicated in apoptosis. We provided direct evidence for HA binding to CD44 in these processes. Namely, mutation of only two amino acids in the ectodomain of CD44 (R78S-Y79F), essential for HA binding [44], sensitized the osteosarcoma cells to DOX treatment (Figure 3). Our results are consistent with previous reports showing that externally added HA polymers exert an inhibitory effect on chemoresistance, by competing with interaction of endogenous HA with its receptors [15,59].

We also showed that the shortest isoforms of CD44, so called CD44s, which does not contain any sequences encoded by alternatively spliced variant exons, was sufficient to confer resistance to doxorubicin in osteosarcoma cells (Figure 2). Nevertheless, as we have shown previously, the *Nf2*-deficient osteosarcomas express additional CD44 splice variants [38]. Thus, we cannot exclude that splice variants of CD44 might regulate additional processes involved in osteosarcoma chemoresistance. A novel finding from our study was that soluble CD44ICD mimicking proteolytic cleavage product regulates MDR1 expression on transcriptional level (Figure 4A,B,D,F). CD44 was shown to modulate transcription of genes containing either CIRE (5′-CCTGCG-3′) or TRE (5′-TGAC/GTCA-3′) sequences in their promoters [24,45,46]. The promoters of *Abcb1a* and *Abcb1b* genes encoding mouse MDR1 protein contain TRE element consensus sequences, providing physical basis for CD44ICD-mediated transcriptional regulation. CD44ICD alone is not sufficient to stimulate transcriptional responses [24], yet it may modulate transcription by affecting other transcription factors or transcriptional coactivators. These CD44ICD collaborators in the regulation of MDR1 expression remain to be identified, but could include Runx2, c-Fos/c-Jun, and CBP/p300 [24,45,47,48].

Doxorubicin belongs to the anthracycline anticancer drug family [2]. The classical mechanism of action by which doxorubicin functions is the interference with Topoisomerase II [2]. This leads to DNA damage in the form of double-strand breaks [2]. As a consequence of double-strand breaks, the DNA damage response and p53 pathways are activated, which lead to cell cycle arrest and cell death [55]. Topoisomerase II is essential for the survival of rapidly dividing cells, such as cancer cells that are more sensitive to DNA breaks than normal quiescent cells [2]. p53-deficient osteosarcoma cells were shown to be resistant to doxorubicin-induced apoptosis [60], highlighting the crucial role of p53 pathway is mediating the antitumoral effects of doxorubicin. Intriguingly, p53 was shown to inhibit CD44 in response to stress conditions by directly binding to its promoter [61]. This suppression of CD44 by p53 was shown to be required for response of untransformed cells to stress-induced, p53-dependent cytostatic and apoptotic signals [61]. These results suggest that the actions of CD44 may otherwise block apoptosis. In the current study, we showed that doxorubicin treatment leads to accumulation of p53 in *Cd44*-negative osteosarcoma cells (Figure 7). *Cd44*-positive osteosarcoma cells did not express significant levels of p53; however, this may result from lower uptake of doxorubicin due to higher expression of MDR1 in those cells. Interestingly, we found that one of the genes regulated by p53, *Perp*, was highly significantly upregulated in *Cd44*-negative mouse osteosarcoma tissues and cells, when compared to their *Cd44*-positive counterparts (Figure 5C and Figure 6). *Perp* is a target of p53 that becomes specifically trans-activated during apoptosis, but not during cell-cycle arrest [49]. These results suggest that doxorubicin-treated cells expressing CD44 might be less susceptible to apoptosis than *Cd44*-negative cells, due to suppression of *Perp*. Moreover, we found that *Cd44*-positive osteosarcoma cells express lower levels of another p53 pathway member, *Mybbp1a* (Figure 6). *Mybbp1a* was shown to interact with p53 and to mediate anoikis in a p53 dependent manner [53]. These finding are very important, as somatic *TP53* mutations belong to the most common genetic alterations in human osteosarcoma and are found in 74–90% of osteosarcoma patients [62,63]. Our studies suggest that targeting CD44-dependent pathways could serve as one option for sensitizing osteosarcoma to chemotherapy.

## 4. Materials and Methods

### 4.1. Antibodies for Western Blotting

Table 1 lists antibodies used for Western Blotting.

### 4.2. Cell Lines

Osteosarcoma cells were derived from tumors generated in *Cd44^+/+^*, *Nf2^Δ2/+^* mice [38]. Establishment of the cell lines was described previously [38]. Briefly, osteosarcoma tissue was washed with PBS and minced into 1–3 mm^3^ pieces with the use of a scalpel. Tissue fragments were plated in DMEM (Life Technologies GmbH, Darmstadt, Germany) supplemented with 10% FBS (PAN-Biotech GmbH, Aidenbach, Germany) and 1% penicillin-streptomycin (Sigma-Aldrich, Taufkirchen, Germany). Outgrowing cells were re-plated on fresh plates. After approximately 12 passages the cultures presented a macroscopically homogenous population of osteosarcoma cells, negative for *Nf2* (characteristic for tumors [38]. Osteosarcoma cells were cultured in DMEM supplemented with 10% FBS and 1% penicillin-streptomycin. For investigation of chemoresistance, the serum concentration was reduced to 7%. All cells were maintained in a humidified atmosphere with 5% CO_2_ at 37 °C. All experiments were performed with mycoplasma-free cells.

### 4.3. DNA Transfections

All DNA transfections were performed in 6-well plates using the liposomal transfection reagent Lipofectamine 2000 (Thermo Fisher Scientific GmbH, Darmstadt, Germany), following the manufacturer’s instructions. 4 μg of DNA was used per transfection.

### 4.4. Genetic Manipulation of CD44 Expression

CRISPR/Cas9 mediated knockout and reconstitution of CD44 expression in *Cd44*-negative osteosarcoma cells were performed as previously described [38]. pSpCas9(BB)-2A-GFP (PX458) was a gift from Feng Zhang (Addgene plasmid #48138). Sequences of primers for targeting exon 1 of the *Cd44* gene: 5′-CACCGAGTTTTGGTGGCACACAGCT-3′ (forward) and 5′-AAACAGCTGTGTGCCACCAAAACTC-3′ (reverse). *Cd44*-negative and GFP-positive single cell clones were selected by flow cytometry (FACSAria) after staining of CD44 with a PE-conjugated CD44 antibody (clone IM7, eBioscience GmbH, Frankfurt am Main, Germany). A hyaluronic acid binding deficient mutant of CD44s was generated by site-directed mutagenesis using the following primer pair: R78S-Y79F forward: 5′-gggtttgaaacatgcAGCTTCgggttcatagaaggac-3′ and R78S-Y79F reverse: 5′-gtccttctatgaacccGAAGCTgcatgtttcaaaccc-3′. Mutations are indicated in capitals. cDNA encoding rat Cd44s, Cd44s-R78S-Y79F-Mt mutant or the GFP-fused, c-Myc-tagged CD44ICD was subcloned into pCDH lentiviral vector. For lentiviral transductions, HEK293 cells were transiently transfected with pCDH plasmid containing the construct of interest, together with packaging and envelope plasmids (pCMVdelR8.91 and pMD.G-VSVG, respectively). The viral particles were harvested 48 h later and filtered through 0.22 µM pore cellulose acetate filters. Filtered supernatants were used for transduction. Cells with CD44 expression similar to wild-type cells were selected by flow cytometry (FACSAria), after staining of CD44 with a PE-conjugated CD44 antibody.

### 4.5. Determination of IC50 of DOX

*Cd44*-positive and *Cd44*-negative osteosarcoma cells were seeded in 96-well plates (5 × 10^3^ cells/well) and allowed to adhere overnight. Doxorubicin was purchased from Enzo Life Sciences GmbH (Lörrach, Germany) and resuspended in deionised water. Cells were incubated with concentrations of doxorubicin ranging between 0.125 μM and 10 μM for 72 h, after which a WST-1 assay was performed according to the manufacturer’s instructions (Roche diagnostics GmbH, Penzberg, Germany). Results of the WST-1 assay were measured at 450 nm using Azure Biosystems microplate reader. All viability assays were performed in quintuplicates.

### 4.6. Colony Formation Assay under Nonadherent Conditions

Osteosarcoma cells were seeded in triplicates at a density 0.1 × 10^5^ cells per well of a Costar 96 well ultra-low attachment surface plate in DMEM supplemented with 10% FBS or without FBS. Doxorubicin was added at a concentration of 1 μM. After 8 days of culture, the viable cells were detected using WST-1 assay according to the manufacturer’s instructions. Absorbance was measured using Azure Biosystems microplate reader. Photographs of the colonies were taken using a Leica DFC290 microscope (Leica Microsystems, Wetzlar, Germany) with 4× magnification. The surface area of photographed colonies was calculated with ImageJ.

### 4.7. Western Blot

Protein levels were determined after 24 h of compound treatment. Cells were treated with 1–1.5 μM doxorubicin (water soluble), 10 µM batimastat (DMSO soluble) (Hölzel Diagnostika Handels GmbH, Köln, Germany) or pure solvents. Cells were harvested and denatured by boiling for 7 min in SDS-PAGE sample buffer containing Halt protease and phosphatase inhibitor cocktail (Life Technologies GmbH, Darmstadt, Germany). Proteins were separated on 8% acrylamide gels by using SDS-PAGE and transferred to nitrocellulose membranes. Blocking was performed for 1 h at room temperature, using 5% low-fat milk powder in TBS containing 0.1% Tween20 solution. Membranes were incubated with primary antibody overnight at 4 °C, followed by HRP-labeled secondary antibody for 1 h at room temperature. 5% BSA in TBS containing 0.1% Tween20 was used as diluent for CD44 antibody clone E7K2Y. All other primary and secondary antibodies were diluted in 5% low-fat milk powder in TBS containing 0.1% Tween20 solution. Blots were developed using ECL solution (Clarity Western ECL Substrate, Bio-Rad Laboratories GmbH, Feldkirchen, Germany). The intensity of immunoblot bands was quantified with ImageJ software.

### 4.8. Real-Time Quantitative Reverse Transcription PCR (Real-Time qRT-PCR)

Total RNA was isolated from cell lines using RNeasy mini kit (Qiagen GmbH, Hilden, Germany) as per manufacturer’s instructions. cDNA was prepared from 1 µg of total RNA using iScript cDNA synthesis kit (Bio-Rad Laboratories GmbH) and used for qRT-PCR reaction at final dilution of 1:75. PCR amplification was performed with iQ^TM^SYBR green supermix (Bio-Rad Laboratories GmbH) on CFX Connect™ real-time PCR detection system. *Hprt1* and *Tbp* housekeeping genes were used as references. Oligonucleotides were used at final concentration of 53 nM. Sequences of oligonucleotides used for real time qRT-PCR are listed in Table 2. Relative expression values were calculated using REST-MCS software [64].

### 4.9. Detection of Putative Transcription Factor Binding Sites

The sequences of mouse *Abcb1a* (ENSMUSG00000040584) and *Abcb1b* (ENSMUSG00000028970) genes were downloaded from Ensembl genome browser. Available online: http://www.ensembl.org/index.html (accessed on 17 June 2022). The promoters, including 1000 nucleotides upstream of the transcription initiation site, were analyzed for presence of 5′-CCTGCG-3′ CIRE and 5′-TGAC/GTCA-3′ TRE elements.

### 4.10. RNA Sequencing and Gene Expression Analysis

Osteosarcoma tissues were derived from *Cd44^+/+^*; *Nf2^Δ2/+^* and *Cd44*^−/−^; *Nf2^Δ2/+^* mice described previously [38]. Total RNA was isolated using RNeasy mini kit (Qiagen) per manufacturer’s instructions. Sequencing of RNA samples was done using Illumina’s next-generation sequencing methodology [65]. In detail, quality check and quantification of total RNA was done using the Agilent Bioanalyzer 2100 in combination with the RNA 6000 nano kit (Agilent Technologies). Library preparation was done using Illumina’s TruSeq stranded mRNA library preparation kit following the manufacturer’s description. Quantification and quality check of libraries was done using the Agilent Bioanalyzer 2100 in combination with the DNA 7500 kit. Libraries were sequenced on a HiSeq2500 running in 51cycle/single-end/high-output mode. Libraries were sequenced in two lanes. Sequence information was extracted in FastQ format using Illumina’s bcl2fastq v.1.8.4. Sequencing resulted in an average of around 24mio reads per sample. Reads were aligned to the mouse reference genome sequence (ENSEMBL Mus musculus GRCm38.84) using HISAT2 (hisat2-2.0.2-beta). Read counts were determined by read summarization with feature Counts (subread-2.0.0). R programming language and related packages were used to further analyze RNA Sequencing data. For principal component analysis (PCA) genes were first subset by removing any genes that were not ‘detectable’ (genes that showed 0 or 1 read counts cumulatively across all samples). Log2-transformed values were calculated from remaining raw count numbers using the rlog function from DESeq2 and used as input for PCA calculated by the PCAtools function in R. Multivariate analysis of gene expression between *Cd44*-positive and *Cd44*-negative primary osteosarcomas was performed using an MADE4 package of R software. Differentially regulated genes were determined using rlog function of DESeq2 package. All genes with Benjamin Hochberg adjusted *p*-value (padj) < 0.05 were included.

### 4.11. Statistical Analysis

For all quantitative analyses, comparisons between groups were made with unpaired Student’s *t*-test unless stated otherwise. Two-tailed *p*-value ≤ 0.05 was considered as significant (n.s.: *p* > 0.05, *: *p* ≤ 0.05, **: *p* ≤ 0.01, ***: *p* ≤ 0.001, ****: *p* ≤ 0.0001).

## Figures and Tables

**Table 1:** Antibodies for Western Blotting.

Name	Cat. No.	Company	Dilution
Actin, clone 2Q1055	sc-58673	Santa Cruz Biotechnology, Inc., Heidelberg, Germany	1:1000
CD44, clone E7K2Y	37259	Cell Signaling Technology, Leiden, The Netherlands	1:1000
c-Myc, clone 9E10	sc-40	Santa Cruz Biotechnology, Inc., Heidelberg, Germany	1:500
GAPDH, clone G-9	sc-365062	Santa Cruz Biotechnology, Inc., Heidelberg, Germany	1:1000
MDR1, clone E1Y7S	13978	Cell Signaling Technology, Leiden, The Netherlands	1:1000
p53 (CM5)	NCL-L-p53-CM5p	Leica Biosystems, Nussloch, Germany	1:2000
p53 (Ser15)	9284	Cell Signaling Technology, Leiden, The Netherlands	1:1000
Cleaved PARP, clone D6X6X	9284	Cell Signaling Technology, Leiden, The Netherlands	1:1000
Tubulin, clone B-5-1-2	T5168	Sigma-Aldrich, Taufkirchen, Germany	1:2000
Goat anti-Mouse IgG (H + L) Secondary Antibody, HRP	31430	Thermo Fisher Scientific GmbH, Darmstadt, Germany	1:1000
Goat anti-Rabbit IgG F(ab′)2 Secondary Antibody, HRP	31461	Thermo Fisher Scientific GmbH, Darmstadt, Germany	1:1000

**Table 2:** Oligonucleotides used for real time qRT-PCR.

Name	Primer Sequence 5′-...............-3′
Hprt1 Fwd1	GTTAAGCAGTACAGCCCCAAA
Hprt1 Rev1	AGGGCATATCCAACAACAAACTT
Tbp Fwd1	GGCCTCTCAGAAGCATCACTA
Tbp Rev1	GCCAAGCCCTGAGCATAA
Bcl2 Fwd1	GACTGAGTACCTGAACCGGC
Bcl2 Rev1	TCACTTGTGGCCCAGGTATG
Appl2 Fwd1	AGATGACACTGGCGGAAGTC
Appl2 Rev1	GCACGTGATTGTCGGTGTTC
Mybbp1a Fwd1	GCACAAGCTGCCTAATGTGG
Mybbp1a Rev1	AGGACGGATTCTTCAGCAGC
Perp Fwd1	GGCCTAATCCCTCCCAACTG
Perp Rev1	TCCTAGGATGTCTGCATGGC
Abcb1b Fwd1	CTTCACCCAGGCCATGATGT
Abcb1b Rev1	GGCACCAAAGACAACAGCAG
Ppp2r5b_Fwd1	TCAGCTGGCATACTGTGTGG
Ppp2r5b_Rev1	CTCTTCCATCTCCCCCAGGA
Erbb3 Fwd1	CCAGCAGCTGAACAAGGGTA
Erbb3 Rev1	GCCAGTAATCGGGGTTGTCA
Lef1 Fwd1	CGGGAAGAGCAGGCCAAATA
Lef1 Rwd1	CGCTGACCAGCCTGGATAAA
CD44all Fwd1	TCTGCCAGGCTTTCAACAGT
CD44all Rev1	CTGCACAGATAGCGTTGGGA

## Data Availability

RNA-sequencing data have been deposited in the Gene Expression Omnibus (GEO) public repository under the accession number GSE210023. The remaining data is contained within the article or supplementary material.

## Supplementary Materials

The following supporting information can be downloaded at: https://www.mdpi.com/article/10.3390/ijms23158616/s1.

## Abbreviations

ABC	ATP-binding cassette
BCRP	breast cancer resistance protein
CD44	cluster of differentiation 44
CD44ICD	intracellular domain of CD44
CIRE	CD44ICD response element
DOX	doxorubicin
ERM	ezrin, radixin, moesin
HA	hyaluronic acid
MDR	multidrug resistance
MRP1	multidrug resistance-associated protein 1
MRP2	multidrug resistance-associated protein 2
NF2	neurofibromatosis type 2
PERP	p53 apoptosis effector related to PMP-22
qRT-PCR	quantitative reverse transcription PCR
TRE	12-O-tetradecanoylphorbol-13-acetate response element
