# Supplementary material for: CD44 Contributes to the Regulation of MDR1 Protein and Doxorubicin Chemoresistance in Osteosarcoma
Source: Int J Mol Sci. 2022 Aug 3;23(15):8616. doi: 10.3390/ijms23158616 (PMC9368984; doi:10.3390/ijms23158616)
Supplement: Supplementary file 1 [file ijms-23-08616-s001.zip › Table S1.pdf]

Table S1. List of genes that are differentially regulated between *Cd44*-positive and *Cd44*-negative osteosarcomas. The genes were determined using rlog function of DESeq2 package. All genes with Benjamin Hochberg adjusted p-value (**padj**) <0.05 were included. The data are represented as Wald-statistic (**stat**). Genes with negative Wald-statistic values are down-regulated in *Cd44*$^{-/-}$ compared to Cd44+/+ primary osteosarcomas and genes with positive values are upregulated.

$$\text{Wald-statistic} = \frac{\text{log2FoldChange}}{\text{standard error of log2FoldChange}}$$

Ensembl_id	Symbol_mouse	*Cd44*$^{+/+}$_*Cd44*$^{-/-}$_padj	*Cd44*$^{+/+}$_*Cd44*$^{-/-}$_stat
ENSMUSG00000036856	Wnt4	4.55634E-16	-9.22454
ENSMUSG00000000244	Tspan32	4.55634E-16	-9.15492
ENSMUSG00000079608	Stard6	5.76623E-10	-7.39855
ENSMUSG00000078949	R3hdml	1.44812E-09	-7.24507
ENSMUSG00000074782	4833422C13Rik	9.637E-09	-6.91733
ENSMUSG00000031169	Porcn	2.55465E-07	-6.36269
ENSMUSG00000032940	Rbm11	1.61262E-06	-6.01381
ENSMUSG00000020263	Appl2	1.61262E-06	-6.01231
ENSMUSG00000025854	Fam20c	1.64308E-06	-6.00095
ENSMUSG00000051890	Klhdc1	2.02747E-06	-5.94928
ENSMUSG00000062743	Zfp677	5.2695E-06	-5.74753
ENSMUSG00000087006	Gm13889	5.41526E-06	-5.73138
ENSMUSG00000034818	Celf5	5.41526E-06	-5.72738
ENSMUSG00000041794	Myrip	2.11687E-05	-5.42839
ENSMUSG00000023990	Tfeb	2.17627E-05	-5.38442
ENSMUSG00000034041	Lyl1	2.67195E-05	-5.32584
ENSMUSG00000023336	Wfdc1	2.68383E-05	-5.32217
ENSMUSG00000053965	Pde5a	3.68243E-05	-5.242
ENSMUSG00000065564	Mirlet7b	3.71134E-05	-5.23294
ENSMUSG00000059708	Akap17b	3.71134E-05	-5.22874
ENSMUSG00000027272	Ubr1	0.00004235	-5.19041
ENSMUSG00000052676	Zmat1	4.37731E-05	-5.17123
ENSMUSG00000043822	Adamtsl5	4.77867E-05	-5.13512
ENSMUSG00000034471	Caskin2	4.85251E-05	-5.12632
ENSMUSG00000027678	Ncoa3	4.95849E-05	-5.11639
ENSMUSG00000022961	Son	0.000051334	-5.10317
ENSMUSG00000042662	Dusp15	6.76031E-05	-5.00282
ENSMUSG00000025701	Alox5	7.92077E-05	-4.95497
ENSMUSG00000024087	Cyp1b1	8.02592E-05	-4.94988
ENSMUSG00000046822	Slc39a3	8.18302E-05	-4.94485
ENSMUSG00000029343	Crybb1	8.84797E-05	-4.92102
ENSMUSG00000029925	Tbxas1	9.04103E-05	-4.91441
ENSMUSG00000037108	Zcwpw1	0.000113423	-4.86389
ENSMUSG00000042751	Nmnat2	0.000117993	-4.8526
ENSMUSG00000035967	Ddx26b	0.000122574	-4.83938
ENSMUSG00000072501	Phf20l1	0.000130872	-4.82412
ENSMUSG00000032796	Lama1	0.000139505	-4.80916
ENSMUSG00000060216	Arrb2	0.000145965	-4.79384
ENSMUSG00000051669	AU021092	0.000145965	-4.79361
ENSMUSG00000039086	Ss18l1	0.000154615	-4.77826
ENSMUSG00000035678	Tnfsf9	0.000156463	-4.77189
ENSMUSG00000021071	Trim9	0.000158234	-4.76798
ENSMUSG00000072653	Zfp783	0.000171283	-4.74792
ENSMUSG00000029544	Cabp1	0.000196135	-4.71215
ENSMUSG00000015981	Stk32c	0.000196135	-4.71152
ENSMUSG00000039908	Slc26a11	0.000209178	-4.68703
ENSMUSG00000022096	Hr	0.000220961	-4.67032
ENSMUSG00000038271	Iffo1	0.000237409	-4.64931
ENSMUSG00000058248	Kcnh1	0.000257732	-4.62227
ENSMUSG00000035722	Abca7	0.000289557	-4.58954
ENSMUSG00000074414	Gm10691	0.000328214	-4.55767
ENSMUSG00000034758	Tle6	0.000360983	-4.53446
ENSMUSG00000020793	Galr2	0.000367826	-4.52893
ENSMUSG00000041515	Irf8	0.00037964	-4.52068
ENSMUSG00000033256	Shf	0.000386787	-4.51518
ENSMUSG00000026883	Dab2ip	0.000395439	-4.50818
ENSMUSG00000087396	4933407K13Rik	0.000435744	-4.48072
ENSMUSG00000098055	Gm26947	0.000441188	-4.4751
ENSMUSG00000000325	Arvcf	0.000458495	-4.46296
ENSMUSG00000025158	Rfng	0.00047348	-4.44916
ENSMUSG00000053687	Dpep2	0.000521707	-4.42231
ENSMUSG00000034037	Fgd5	0.000521707	-4.42097
ENSMUSG00000023912	Slc25a27	0.000521707	-4.42052
ENSMUSG00000020658	Efr3b	0.000525945	-4.417
ENSMUSG00000040276	Pacsin1	0.000541683	-4.40709
ENSMUSG00000036026	Tmem63b	0.00055486	-4.40121
ENSMUSG00000028078	Dclk2	0.000604292	-4.37868
ENSMUSG00000075324	Fign	0.000604795	-4.37784
ENSMUSG00000071192	Wfikkn1	0.000613462	-4.37408
ENSMUSG00000072844	G530011O06Rik	0.000642993	-4.3625
ENSMUSG00000033705	Stard9	0.000646728	-4.35993
ENSMUSG00000021478	Drd1a	0.000690549	-4.34297

ENSMUSG00000015396	Cd83	0.000710422	-4.33481
ENSMUSG00000081044	AU015836	0.00074017	-4.32334
ENSMUSG00000032702	Kank1	0.00074017	-4.32323
ENSMUSG00000099021	Metazoa_SRP	0.000746901	-4.31974
ENSMUSG00000047238	Mageh1	0.000779679	-4.30735
ENSMUSG00000028266	Lmo4	0.000832574	-4.28706
ENSMUSG00000034771	Tle2	0.000884565	-4.26951
ENSMUSG00000032017	Grik4	0.000947614	-4.24935
ENSMUSG00000022098	Bmp1	0.000952302	-4.24766
ENSMUSG00000068263	Efcc1	0.000969774	-4.24299
ENSMUSG00000022243	Slc45a2	0.000999804	-4.23205
ENSMUSG00000048827	Pkd1l3	0.00102359	-4.2256
ENSMUSG00000039904	Gpr37	0.00105223	-4.21881
ENSMUSG00000052504	Epha3	0.00113878	-4.19693
ENSMUSG00000097085	Gm26634	0.00118037	-4.1844
ENSMUSG00000063727	Tnfrsf11b	0.00119852	-4.17969
ENSMUSG00000043673	Kcns3	0.00121371	-4.17627
ENSMUSG00000022657	Cd96	0.00124126	-4.16949
ENSMUSG00000025648	Pfkfb4	0.00142081	-4.12718
ENSMUSG00000026458	Ppfia4	0.00142652	-4.12572
ENSMUSG00000022974	Paxbp1	0.00142667	-4.12464
ENSMUSG00000068373	D430041D05Rik	0.00144697	-4.11981
ENSMUSG00000020954	Strn3	0.00151498	-4.10816
ENSMUSG00000022987	Zfp641	0.00156039	-4.09821
ENSMUSG00000035681	Kcnc2	0.00156752	-4.09663
ENSMUSG00000034799	Unc13a	0.00156975	-4.09579
ENSMUSG00000017734	Dbndd2	0.00159425	-4.09117
ENSMUSG00000016933	Plcg1	0.00161538	-4.08741
ENSMUSG00000025092	Hspa12a	0.00161538	-4.08709
ENSMUSG00000030557	Mef2a	0.00172288	-4.06955
ENSMUSG00000030306	Tmtc1	0.00175337	-4.06496
ENSMUSG00000026062	Slc9a2	0.00185457	-4.04931
ENSMUSG00000051723	Rpl31-ps13	0.00185662	-4.04805
ENSMUSG00000044055	Otos	0.00195583	-4.03135
ENSMUSG00000074676	Foxs1	0.00198158	-4.0263
ENSMUSG00000032006	Pdgfd	0.00198708	-4.02467
ENSMUSG00000079056	Kcnip3	0.00216546	-4.00146
ENSMUSG00000019894	Slc6a15	0.00223037	-3.9935
ENSMUSG00000067942	Zfp160	0.00240798	-3.97192
ENSMUSG00000004788	Eif2b2	0.00248268	-3.9632
ENSMUSG00000024013	Fgd2	0.00269003	-3.941
ENSMUSG00000041193	Pla2g5	0.00274414	-3.93414
ENSMUSG00000039873	Neurl2	0.00278337	-3.92685
ENSMUSG00000008999	Bmp7	0.00278592	-3.92616
ENSMUSG00000032717	Mdfi	0.00282264	-3.92208
ENSMUSG00000034111	Tmed8	0.00285483	-3.91797
ENSMUSG00000024008	Cpne5	0.00285542	-3.91746
ENSMUSG00000061898	Rbak	0.00287656	-3.91522
ENSMUSG00000042312	S100a13	0.00293659	-3.9084
ENSMUSG00000079429	Mroh2a	0.00295506	-3.90598
ENSMUSG00000034157	2310044G17Rik	0.00297383	-3.90399
ENSMUSG00000097494	4933406C10Rik	0.0029813	-3.90253
ENSMUSG00000037860	Aim2	0.0029813	-3.90248
ENSMUSG00000021567	Nkd2	0.00301284	-3.89948
ENSMUSG00000098495	RP24-113D21.1	0.00301516	-3.89884
ENSMUSG00000003378	Grik5	0.00303305	-3.89641
ENSMUSG00000063415	Cyp26b1	0.00303305	-3.89518
ENSMUSG00000027620	Rbm39	0.00322312	-3.87817
ENSMUSG00000033972	Zfp944	0.00351889	-3.85276
ENSMUSG00000038055	Dexi	0.00357526	-3.84668
ENSMUSG00000042109	Csdc2	0.00363438	-3.84135
ENSMUSG00000091530	Cldn20	0.0038001	-3.82779
ENSMUSG00000037336	Mfsd2b	0.00391974	-3.81928
ENSMUSG00000020773	Trim47	0.00421804	-3.7986
ENSMUSG00000066705	Fxyd6	0.0042413	-3.79635
ENSMUSG00000022914	Brwd1	0.0042413	-3.79634
ENSMUSG00000091115	BC027582	0.00428981	-3.7931
ENSMUSG00000025423	Pias2	0.00435248	-3.78864
ENSMUSG00000070526	Peg12	0.00441646	-3.78459
ENSMUSG00000090863	A530084C06Rik	0.00444785	-3.7824
ENSMUSG00000021222	Dcaf4	0.00445985	-3.78088
ENSMUSG00000061462	Obscn	0.0045912	-3.77195
ENSMUSG00000022468	Endou	0.00464954	-3.76775

ENSMUSG00000093459	5730480H06Rik	0.00464954	-3.76754
ENSMUSG00000061689	Dlgap4	0.0047664	-3.75799
ENSMUSG00000042063	Zfp386	0.00478934	-3.75633
ENSMUSG00000014361	Mertk	0.00478934	-3.75596
ENSMUSG00000056270	Prr9	0.00480939	-3.75408
ENSMUSG00000052160	Pld4	0.00483103	-3.75213
ENSMUSG00000039483	Asb6	0.00508885	-3.7362
ENSMUSG00000021200	Asb2	0.00543745	-3.7126
ENSMUSG00000022973	Synj1	0.00544435	-3.71151
ENSMUSG00000029919	Hpgds	0.00554014	-3.70547
ENSMUSG00000033192	Lpcat2	0.0056818	-3.69828
ENSMUSG00000025432	Avil	0.00573256	-3.69483
ENSMUSG00000031216	Stard8	0.00590249	-3.68386
ENSMUSG00000027896	Slc16a4	0.00591653	-3.68247
ENSMUSG00000032948	Lipi	0.00598538	-3.67701
ENSMUSG00000055436	Srsf11	0.00598538	-3.67628
ENSMUSG00000067713	Prkag1	0.00615148	-3.66712
ENSMUSG00000022469	Rapgef3	0.00619277	-3.66503
ENSMUSG00000020151	Ptprr	0.00629797	-3.66033
ENSMUSG00000024462	Gabbr1	0.00638423	-3.65613
ENSMUSG00000071103	1700029J07Rik	0.00638423	-3.65608
ENSMUSG00000053117	E330013P04Rik	0.00645409	-3.65253
ENSMUSG00000054737	Zfp182	0.00650481	-3.65013
ENSMUSG00000029924	Slc37a3	0.00664862	-3.64305
ENSMUSG00000046415	B430212C06Rik	0.00664862	-3.64262
ENSMUSG00000035597	Prpf39	0.00690604	-3.63056
ENSMUSG00000049521	Cdc42ep1	0.00695947	-3.62746
ENSMUSG00000033147	Slc22a15	0.00716554	-3.61766
ENSMUSG00000034789	Rab24	0.00727727	-3.61253
ENSMUSG00000021224	Numb	0.0074605	-3.60497
ENSMUSG00000029490	Mfsd7a	0.00754677	-3.60027
ENSMUSG00000021259	Cyp46a1	0.00754677	-3.60013
ENSMUSG00000025145	Lrrc45	0.00765412	-3.59426
ENSMUSG00000025572	Tmc6	0.00772318	-3.59119
ENSMUSG00000043165	Lor	0.0077254	-3.59075
ENSMUSG00000033540	Idua	0.007809	-3.58758
ENSMUSG00000020262	Adarb1	0.00814139	-3.57342
ENSMUSG00000027082	Tfpi	0.00820581	-3.56992
ENSMUSG00000051373	Ppapdc3	0.00822238	-3.56903
ENSMUSG00000028476	Reck	0.0082774	-3.56692
ENSMUSG00000074918	5430417L22Rik	0.00828309	-3.56629
ENSMUSG00000087187	Gm13431	0.00828309	-3.56582
ENSMUSG00000050373	Snx21	0.00836152	-3.56248
ENSMUSG00000019790	Stxbp5	0.00844563	-3.55879
ENSMUSG00000029601	Iqcd	0.00853554	-3.55529
ENSMUSG00000013858	Tmem259	0.00857276	-3.55344
ENSMUSG00000041623	D11Wsu47e	0.00868826	-3.54916
ENSMUSG00000017639	Rab11fip4	0.00869662	-3.5479
ENSMUSG00000022454	Nell2	0.00884147	-3.54249
ENSMUSG00000049409	Prokr1	0.00895697	-3.53731
ENSMUSG00000022434	Fam118a	0.00898828	-3.53604
ENSMUSG00000002908	Kcnn1	0.00918715	-3.52844
ENSMUSG00000015702	Anxa9	0.00919143	-3.5277
ENSMUSG00000022489	Pde1b	0.00933039	-3.52269
ENSMUSG00000027776	Il12a	0.00941567	-3.5199
ENSMUSG00000028134	Ptbp2	0.00941567	-3.51958
ENSMUSG00000071648	Rom1	0.00959696	-3.51177
ENSMUSG00000027848	Olfml3	0.00961814	-3.51016
ENSMUSG00000048200	Efcab4a	0.00983248	-3.50293
ENSMUSG00000040254	Sema3d	0.00984707	-3.502
ENSMUSG00000021704	Mtx3	0.00984707	-3.50186
ENSMUSG00000041115	Iqsec2	0.00996473	-3.49737
ENSMUSG00000059327	Eda	0.00996473	-3.49734
ENSMUSG00000031145	Prickle3	0.0102913	-3.48603
ENSMUSG00000042607	Asb4	0.0103287	-3.48439
ENSMUSG00000005087	Cd44	0.0103465	-3.4836
ENSMUSG00000087064	Gm11721	0.0104386	-3.48056
ENSMUSG00000047117	Ankdd1b	0.0106492	-3.47454
ENSMUSG00000039166	Akap7	0.0106728	-3.47361
ENSMUSG00000085174	Gm16206	0.0110268	-3.46305
ENSMUSG00000068289	Cma2	0.0112343	-3.45585
ENSMUSG00000028108	Ecm1	0.0114718	-3.44841
ENSMUSG00000030761	Myo7a	0.0117753	-3.4392

ENSMUSG00000015852	Fcrls	0.0117753	-3.43878
ENSMUSG00000036196	Slc26a8	0.0117839	-3.43803
ENSMUSG00000024777	Ppp2r5b	0.0122754	-3.42189
ENSMUSG00000032633	Flcn	0.012364	-3.41759
ENSMUSG00000006445	Epha2	0.0125956	-3.41112
ENSMUSG00000062866	Phactr2	0.013	-3.40074
ENSMUSG00000014303	Glis2	0.0131219	-3.39756
ENSMUSG00000035891	Cerk	0.0132971	-3.39253
ENSMUSG00000082193	Rpl5-ps1	0.0133399	-3.39116
ENSMUSG00000022197	Pdzd2	0.0133692	-3.38837
ENSMUSG00000026640	Plxna2	0.0136144	-3.38152
ENSMUSG00000005057	Sh2b2	0.0136733	-3.37955
ENSMUSG00000060548	Tnfrsf19	0.0136943	-3.37809
ENSMUSG00000027784	Ppm1l	0.0138053	-3.37491
ENSMUSG00000022604	Cep97	0.0138279	-3.37385
ENSMUSG00000047746	Fbxo40	0.014113	-3.36761
ENSMUSG00000004187	Kifc2	0.0142481	-3.36451
ENSMUSG00000023021	Cers5	0.0142481	-3.36334
ENSMUSG00000035245	Eogt	0.0142481	-3.36299
ENSMUSG00000057219	Armc7	0.0142481	-3.36271
ENSMUSG00000048699	4732456N10Rik	0.0143101	-3.36104
ENSMUSG00000097033	Gm26819	0.014489	-3.357
ENSMUSG00000038210	Hoxa11	0.0146822	-3.35123
ENSMUSG00000035776	Cd99l2	0.0146822	-3.35115
ENSMUSG00000039810	Zc3h10	0.0147188	-3.34902
ENSMUSG00000024299	Adamts10	0.0148003	-3.34719
ENSMUSG00000079414	Gm11110	0.014825	-3.34638
ENSMUSG00000022999	Lmbr1l	0.014825	-3.34611
ENSMUSG00000086839	Gm11973	0.014825	-3.34571
ENSMUSG00000000392	Fap	0.0148837	-3.34414
ENSMUSG00000021223	Papln	0.0149474	-3.34146
ENSMUSG00000040957	Cables1	0.0149781	-3.3403
ENSMUSG00000095134	Gm21857	0.0150843	-3.33774
ENSMUSG00000036249	Rbm43	0.0151021	-3.33712
ENSMUSG00000034781	Gna11	0.0151044	-3.33678
ENSMUSG00000021504	B4galt7	0.0151674	-3.33533
ENSMUSG00000096687	AA474331	0.015268	-3.3326
ENSMUSG00000020802	Ube2o	0.015318	-3.33114
ENSMUSG00000009210	2310007L24Rik	0.0158061	-3.32089
ENSMUSG00000095440	Fignl2	0.0159213	-3.31857
ENSMUSG00000026810	Dpm2	0.0159326	-3.31808
ENSMUSG00000050382	Kif7	0.0161125	-3.31348
ENSMUSG00000053957	Gm12474	0.016223	-3.30984
ENSMUSG00000028086	Fbxw7	0.016223	-3.30982
ENSMUSG00000093629	Gm19357	0.0162405	-3.30884
ENSMUSG00000047420	Fam180a	0.0162818	-3.30736
ENSMUSG00000002885	Cd97	0.0162942	-3.30686
ENSMUSG00000017760	Ctsa	0.0163154	-3.30591
ENSMUSG00000027985	Lef1	0.0163291	-3.30496
ENSMUSG00000034881	Tbxa2r	0.0163291	-3.30482
ENSMUSG00000026358	Rgs1	0.0171154	-3.28903
ENSMUSG00000044320	1700001O22Rik	0.0172718	-3.28504
ENSMUSG00000060798	Intu	0.017306	-3.28378
ENSMUSG00000044770	Scml4	0.017532	-3.27912
ENSMUSG00000026930	Gpsm1	0.0178032	-3.27421
ENSMUSG00000064057	Scgb3a1	0.0180298	-3.2694
ENSMUSG00000097488	4732487G21Rik	0.018235	-3.26446
ENSMUSG00000040687	Madd	0.018235	-3.26433
ENSMUSG00000000266	Mid2	0.0183752	-3.26104
ENSMUSG00000056679	Gpr173	0.0185386	-3.25769
ENSMUSG00000023886	Smoc2	0.018551	-3.25626
ENSMUSG00000020009	Ifngr1	0.0186574	-3.25278
ENSMUSG00000093561	Gm20699	0.0186771	-3.25168
ENSMUSG00000032726	Bmp8a	0.0187351	-3.25041
ENSMUSG00000031367	Ap1s2	0.0188121	-3.24857
ENSMUSG00000027329	Spef1	0.0188121	-3.24853
ENSMUSG00000087644	Gm14703	0.0190237	-3.24425
ENSMUSG00000044164	Rnf182	0.0191632	-3.24079
ENSMUSG00000073422	H2-Ke6	0.0191698	-3.24042
ENSMUSG00000034614	Pik3ip1	0.0192482	-3.23898
ENSMUSG00000033765	Calm4	0.0192711	-3.23837
ENSMUSG00000047747	Rnf150	0.0194835	-3.23361
ENSMUSG00000060336	Zfp937	0.0196959	-3.22861

ENSMUSG00000056947	Mab21l1	0.0198507	-3.22475
ENSMUSG00000022996	Wnt10b	0.0200631	-3.22117
ENSMUSG00000028012	Rrh	0.0204749	-3.21442
ENSMUSG00000075590	Nrbp2	0.0204749	-3.21426
ENSMUSG00000086560	Gm13372	0.0205146	-3.21344
ENSMUSG00000000693	Loxl3	0.0206011	-3.21116
ENSMUSG00000022601	Rpl24	0.0206516	-3.20966
ENSMUSG00000041718	Alg13	0.0208989	-3.2049
ENSMUSG00000070891	Gm12689	0.0209196	-3.20435
ENSMUSG00000003178	Mical3	0.0210564	-3.20154
ENSMUSG00000037661	Gpr160	0.0210564	-3.20089
ENSMUSG00000036617	Etl4	0.0211751	-3.19842
ENSMUSG00000030083	Abtb1	0.0214848	-3.19296
ENSMUSG00000068923	Syt11	0.0217613	-3.18743
ENSMUSG00000038178	Slc43a2	0.0219247	-3.18444
ENSMUSG00000040459	Arglu1	0.0221463	-3.18053
ENSMUSG00000027716	Trpc3	0.0224162	-3.17623
ENSMUSG00000040272	Accs	0.0226634	-3.17279
ENSMUSG00000064340	mt-Tl1	0.0226653	-3.17237
ENSMUSG00000085307	Gm11525	0.0226653	-3.17225
ENSMUSG00000020577	Tspan13	0.022911	-3.16746
ENSMUSG00000039496	Cdnf	0.0229946	-3.16572
ENSMUSG00000052336	Cx3cr1	0.0230028	-3.16536
ENSMUSG00000042498	D330045A20Rik	0.0233842	-3.16006
ENSMUSG00000006476	Nsmf	0.0234006	-3.1596
ENSMUSG00000004842	Pou1f1	0.0234706	-3.15744
ENSMUSG00000038564	Ift172	0.0236059	-3.15423
ENSMUSG00000064372	mt-Tp	0.0237247	-3.15213
ENSMUSG00000028681	Ptch2	0.0237929	-3.15077
ENSMUSG00000067931	Zfp948	0.0237929	-3.15048
ENSMUSG00000066682	Pilrb2	0.0240216	-3.14709
ENSMUSG00000003581	Rnf215	0.0246698	-3.13802
ENSMUSG00000072476	Gm9008	0.0249348	-3.13388
ENSMUSG00000037408	Cnnm4	0.0250551	-3.13196
ENSMUSG00000020092	Pald1	0.0252991	-3.12763
ENSMUSG00000045980	Tmem104	0.0252991	-3.12761
ENSMUSG00000013076	Amotl1	0.0253417	-3.12685
ENSMUSG00000053580	Tanc2	0.0256117	-3.12303
ENSMUSG00000019143	Hars2	0.0256117	-3.12287
ENSMUSG00000034168	Irf2bpl	0.0256117	-3.12278
ENSMUSG00000039543	Ttc18	0.0256117	-3.12273
ENSMUSG00000040481	Bptf	0.0257805	-3.12054
ENSMUSG00000027276	Jag1	0.0258496	-3.1195
ENSMUSG00000028909	Ptpru	0.0259612	-3.11763
ENSMUSG00000071604	Fam189a2	0.026012	-3.1164
ENSMUSG00000059866	Tnip2	0.0261588	-3.11424
ENSMUSG00000039850	Endov	0.0266624	-3.10742
ENSMUSG00000082604	Gm8482	0.0266624	-3.10736
ENSMUSG00000097929	2810011L19Rik	0.0268012	-3.10533
ENSMUSG00000051510	Mafg	0.0273464	-3.09837
ENSMUSG00000002428	Hltf	0.0275124	-3.09537
ENSMUSG00000031144	Syp	0.0275318	-3.09488
ENSMUSG00000064927	Gm22043	0.0275404	-3.09443
ENSMUSG00000024287	Thoc1	0.0275404	-3.09405
ENSMUSG00000054580	Pla2r1	0.0281518	-3.08679
ENSMUSG00000012640	Zfp715	0.0282351	-3.08566
ENSMUSG00000034748	Sirt6	0.0284522	-3.08264
ENSMUSG00000022964	Tmem50b	0.0286408	-3.08004
ENSMUSG00000087017	4930417H01Rik	0.0287358	-3.07821
ENSMUSG00000022510	Trp63	0.0293127	-3.07008
ENSMUSG00000029819	Npy	0.02941	-3.06878
ENSMUSG00000027575	Arfgap1	0.0298274	-3.06408
ENSMUSG00000086148	Gm15271	0.0299561	-3.06152
ENSMUSG00000095687	Rnaset2a	0.0299561	-3.0614
ENSMUSG00000084885	3010001F23Rik	0.0300421	-3.06029
ENSMUSG00000019822	Smpd2	0.0300899	-3.05913
ENSMUSG00000023092	Fhl1	0.0301857	-3.05717
ENSMUSG00000025856	Pdgfa	0.0304829	-3.05302
ENSMUSG00000042599	Jhdm1d	0.0304885	-3.05273
ENSMUSG00000021275	Tecpr2	0.0307961	-3.04912
ENSMUSG00000016087	Fli1	0.0307961	-3.04899
ENSMUSG00000019969	Psen1	0.0308789	-3.0474
ENSMUSG00000061758	Akr1b10	0.0308789	-3.04722

ENSMUSG00000031214	Ophn1	0.031218	-3.04311
ENSMUSG00000053702	Nebl	0.031218	-3.04298
ENSMUSG00000097579	Gm26799	0.0315532	-3.03881
ENSMUSG00000026857	Ntmt1	0.031818	-3.03605
ENSMUSG00000031129	Slc9a9	0.0318726	-3.03505
ENSMUSG00000020747	2310067B10Rik	0.0318726	-3.03482
ENSMUSG00000066613	Zfp932	0.0320132	-3.03301
ENSMUSG00000050931	Sgms2	0.0320149	-3.03276
ENSMUSG00000029009	Mthfr	0.0321132	-3.03112
ENSMUSG00000034801	Sos2	0.0322196	-3.02988
ENSMUSG00000046743	Fat4	0.0322423	-3.02936
ENSMUSG00000030247	Kcnj8	0.0326009	-3.02453
ENSMUSG00000033470	Cysltr2	0.0326009	-3.02443
ENSMUSG00000085862	Gm13483	0.0328497	-3.02095
ENSMUSG00000023755	Rhebl1	0.0328992	-3.02016
ENSMUSG00000067101	1700010H22Rik	0.0331493	-3.0172
ENSMUSG00000046982	Tshz1	0.0333991	-3.01381
ENSMUSG00000037013	Ss18	0.0338084	-3.00878
ENSMUSG00000095562	Gm21887	0.0338084	-3.00824
ENSMUSG00000047789	Slc38a9	0.0342721	-3.0034
ENSMUSG00000051786	Tubgcp6	0.0343635	-3.00235
ENSMUSG00000013236	Ptprs	0.0344095	-3.00171
ENSMUSG00000040563	BC018242	0.0347699	-2.99784
ENSMUSG00000042826	Fgf11	0.0347878	-2.99724
ENSMUSG00000035595	1600002K03Rik	0.0347878	-2.99711
ENSMUSG00000097660	Gm26762	0.0347878	-2.99682
ENSMUSG00000047182	Irs3	0.0347935	-2.99624
ENSMUSG00000042286	Stab1	0.0348854	-2.99497
ENSMUSG00000054675	Tmem119	0.0349968	-2.99326
ENSMUSG00000015476	Prrt1	0.0349968	-2.99308
ENSMUSG00000020982	Nemf	0.0353392	-2.98905
ENSMUSG00000029352	Crybb3	0.0353392	-2.98881
ENSMUSG00000024784	Gpha2	0.0353392	-2.98876
ENSMUSG00000031736	4933436C20Rik	0.0353752	-2.9876
ENSMUSG00000033207	Mamdc2	0.0353752	-2.98749
ENSMUSG00000066892	Fbxl12	0.0355418	-2.98572
ENSMUSG00000027452	Acss1	0.0360796	-2.97828
ENSMUSG00000028017	Egf	0.0360796	-2.97826
ENSMUSG00000090015	Gm15446	0.036354	-2.97534
ENSMUSG00000043557	Mdga1	0.0364427	-2.97394
ENSMUSG00000030882	Dnhd1	0.0366143	-2.972
ENSMUSG00000039568	Ubald1	0.0367554	-2.97029
ENSMUSG00000054708	Ankrd24	0.0367768	-2.96988
ENSMUSG00000041479	Syt15	0.03686	-2.96848
ENSMUSG00000025964	Adam23	0.0369228	-2.96749
ENSMUSG00000047250	Ptgs1	0.037017	-2.9655
ENSMUSG00000017765	Slc12a4	0.0370313	-2.965
ENSMUSG00000037965	Zc3h7a	0.0370313	-2.96483
ENSMUSG00000097227	F630040L22Rik	0.0370602	-2.96437
ENSMUSG00000021027	Ralgapa1	0.0370936	-2.96387
ENSMUSG00000032172	Olfm2	0.0375647	-2.95931
ENSMUSG00000097364	Gm26719	0.0377138	-2.95742
ENSMUSG00000036264	Fstl4	0.0382445	-2.95221
ENSMUSG00000024402	Lta	0.0384716	-2.94956
ENSMUSG00000037773	Pced1a	0.0384716	-2.94924
ENSMUSG00000026860	Sh3glb2	0.0384716	-2.94922
ENSMUSG00000009739	Pou6f1	0.0384716	-2.94904
ENSMUSG00000045466	Zfp956	0.0388941	-2.94522
ENSMUSG00000043398	Gpr135	0.038924	-2.94476
ENSMUSG00000051000	Fam160a1	0.0390047	-2.9439
ENSMUSG00000095040	1700001J03Rik	0.0392416	-2.94122
ENSMUSG00000040054	Baz2a	0.0392416	-2.94091
ENSMUSG00000020232	Hmg20b	0.0394598	-2.93875
ENSMUSG00000020099	Unc5b	0.0396168	-2.9373
ENSMUSG00000024524	Gnal	0.0396924	-2.93648
ENSMUSG00000034265	Zdhhc14	0.0397358	-2.93592
ENSMUSG00000070390	Nlrp1b	0.0397424	-2.93556
ENSMUSG00000027422	Rrbp1	0.0397424	-2.93525
ENSMUSG00000022494	Shisa9	0.0402149	-2.93088
ENSMUSG00000032323	Cyp11a1	0.0402476	-2.93025
ENSMUSG00000045008	9030612E09Rik	0.0402476	-2.93019
ENSMUSG00000064369	mt-Te	0.0403565	-2.92912
ENSMUSG00000039193	Nlrc4	0.0403565	-2.92848

ENSMUSG00000027692	Tnik	0.0406963	-2.92542
ENSMUSG00000038671	Arfrp1	0.0407488	-2.9248
ENSMUSG00000005823	Gpr108	0.0410449	-2.92145
ENSMUSG00000024137	E4f1	0.041221	-2.91977
ENSMUSG00000038022	Fam188b	0.0412749	-2.91871
ENSMUSG00000038248	Sobp	0.0412749	-2.91861
ENSMUSG00000090628	Gm17083	0.0414452	-2.91637
ENSMUSG00000086544	Gm13704	0.0420685	-2.91049
ENSMUSG00000037106	Fer1l6	0.0423475	-2.90811
ENSMUSG00000097819	Gm26813	0.0427848	-2.90306
ENSMUSG00000071267	Zfp942	0.0427848	-2.90303
ENSMUSG00000081043	Gm11512	0.0432124	-2.89884
ENSMUSG00000034160	Ogt	0.0432999	-2.89799
ENSMUSG00000038042	Ptpdc1	0.0435529	-2.89584
ENSMUSG00000031217	Efnb1	0.0436666	-2.89469
ENSMUSG00000002668	Dennd1c	0.0440039	-2.89185
ENSMUSG00000022621	Rabl2	0.0441268	-2.89054
ENSMUSG00000003283	Hck	0.0441861	-2.8899
ENSMUSG00000007033	Hspa1l	0.0441982	-2.88939
ENSMUSG00000051228	Nyx	0.0443799	-2.88767
ENSMUSG00000052783	Grk4	0.0443938	-2.88735
ENSMUSG00000064342	mt-Ti	0.044596	-2.88484
ENSMUSG00000097101	1810034E14Rik	0.0447099	-2.88213
ENSMUSG00000017664	Slc35c2	0.044934	-2.88009
ENSMUSG00000085088	4931413K12Rik	0.044934	-2.87994
ENSMUSG00000035697	Hmha1	0.04503	-2.879
ENSMUSG00000042903	Foxo4	0.0451109	-2.87804
ENSMUSG00000050910	Cdr2l	0.0451164	-2.87778
ENSMUSG00000033713	Foxn3	0.0452191	-2.87602
ENSMUSG00000095041	AC149090.1	0.0452191	-2.87554
ENSMUSG00000034165	Ccnd3	0.0453598	-2.87397
ENSMUSG00000024617	Camk2a	0.0454048	-2.87345
ENSMUSG00000028917	Plekhm2	0.0454758	-2.87242
ENSMUSG00000032621	Srek1	0.0456533	-2.86947
ENSMUSG00000058715	Fcer1g	0.0456533	-2.86857
ENSMUSG00000094724	Rnaset2b	0.0456533	-2.86815
ENSMUSG00000066975	Cryba4	0.0456866	-2.86751
ENSMUSG00000040479	Dgkz	0.0457084	-2.86652
ENSMUSG00000085760	Gm14302	0.0457084	-2.86637
ENSMUSG00000081137	BC022960	0.0457084	-2.86631
ENSMUSG00000096606	Gm4980	0.0458176	-2.86463
ENSMUSG00000053178	Gm9897	0.0458176	-2.86435
ENSMUSG00000026867	Gapvd1	0.0458176	-2.86411
ENSMUSG00000029720	Gm20605	0.0458709	-2.86353
ENSMUSG00000048277	Syngr2	0.0460342	-2.86158
ENSMUSG00000029524	Sirt4	0.046107	-2.86011
ENSMUSG00000053436	Mapk14	0.046107	-2.86004
ENSMUSG00000044092	C130050O18Rik	0.0464079	-2.85757
ENSMUSG00000043541	Casc1	0.0466684	-2.85481
ENSMUSG00000041592	Sdk2	0.0466893	-2.85441
ENSMUSG00000090952	Gm17251	0.0471886	-2.85021
ENSMUSG00000024566	Atp9b	0.0472007	-2.84993
ENSMUSG00000024897	Apba1	0.0475094	-2.84724
ENSMUSG00000036560	Lgi4	0.0476361	-2.84618
ENSMUSG00000048442	Smim5	0.0477038	-2.84553
ENSMUSG00000045048	Gm9795	0.047982	-2.84286
ENSMUSG00000064343	mt-Tq	0.0483728	-2.83966
ENSMUSG00000057606	Colq	0.0486109	-2.83681
ENSMUSG00000075511	1700001L05Rik	0.0486121	-2.83646
ENSMUSG00000044857	Lemd2	0.0486918	-2.83573
ENSMUSG00000019880	Rspo3	0.0487347	-2.83504
ENSMUSG00000027474	Ccm2l	0.0489372	-2.8321
ENSMUSG00000045838	A430105I19Rik	0.0490752	-2.83059
ENSMUSG00000041354	Rgl2	0.0490789	-2.83037
ENSMUSG00000029647	Pan3	0.0490995	-2.83003
ENSMUSG00000036890	Gtdc1	0.0498945	2.82408
ENSMUSG00000001417	Rhbg	0.0498389	2.82464
ENSMUSG00000060183	Cxcl11	0.049492	2.82708
ENSMUSG00000025137	Pcyt2	0.0491242	2.82967
ENSMUSG00000052997	Uba2	0.0490361	2.83105
ENSMUSG00000048371	Pdp2	0.0489809	2.83161
ENSMUSG00000020068	Pbld2	0.0488781	2.83269
ENSMUSG00000053644	Aldh7a1	0.0488742	2.83291

ENSMUSG00000032370	Lactb	0.0488742	2.83301
ENSMUSG00000045932	Ifit2	0.0488326	2.83359
ENSMUSG00000031886	Ces2e	0.0488326	2.83365
ENSMUSG00000029012	Orc5	0.0488326	2.83376
ENSMUSG00000086771	1700080G11Rik	0.0488326	2.83418
ENSMUSG00000020990	Cdkl1	0.0487228	2.83532
ENSMUSG00000029151	Slc30a3	0.0486109	2.83667
ENSMUSG00000006517	Mvd	0.0486109	2.83686
ENSMUSG00000087241	Gm11844	0.0484679	2.83822
ENSMUSG00000041360	D19Bwg1357e	0.0484679	2.83826
ENSMUSG00000068874	Selenbp1	0.0484679	2.83855
ENSMUSG00000060988	Galnt13	0.0484679	2.83876
ENSMUSG00000091549	Gm6548	0.0481965	2.84103
ENSMUSG00000032216	Nedd4	0.0480494	2.84221
ENSMUSG00000009681	Bcr	0.0479729	2.84312
ENSMUSG00000026786	Apbb1ip	0.0479427	2.84353
ENSMUSG00000091747	D17H6S56E-5	0.0477461	2.84504
ENSMUSG00000020541	Tom1l1	0.0473441	2.84855
ENSMUSG00000056367	Actr3b	0.0473006	2.84905
ENSMUSG00000084159	Gm12696	0.0471886	2.85041
ENSMUSG00000038576	Susd4	0.0468328	2.85303
ENSMUSG00000016024	Lbp	0.0468328	2.85321
ENSMUSG00000021876	Rnase4	0.0466684	2.85476
ENSMUSG00000042436	Mfap4	0.0466684	2.85496
ENSMUSG00000048644	Ctxn1	0.0466022	2.85583
ENSMUSG00000026317	Cln8	0.046411	2.85734
ENSMUSG00000030754	Copb1	0.0463313	2.8583
ENSMUSG00000023262	Acy1	0.046107	2.8602
ENSMUSG00000056383	AI987944	0.046085	2.86081
ENSMUSG00000038422	Hdhd3	0.046085	2.86088
ENSMUSG00000023906	Cldn6	0.0460342	2.86158
ENSMUSG00000086071	Gm12354	0.0460342	2.86172
ENSMUSG00000025933	Tmem14a	0.0459269	2.86294
ENSMUSG00000022237	Ankrd33b	0.0458176	2.8641
ENSMUSG00000030983	Bccip	0.0458176	2.86443
ENSMUSG00000026170	Cyp27a1	0.0458176	2.86466
ENSMUSG00000032279	Idh3a	0.0458176	2.8651
ENSMUSG00000025785	Exosc7	0.0457084	2.8667
ENSMUSG00000021981	Cab39l	0.0457084	2.86689
ENSMUSG00000033352	Map2k4	0.0456866	2.86758
ENSMUSG00000028480	Glipr2	0.0456533	2.86819
ENSMUSG00000092116	Gm10320	0.0456533	2.86832
ENSMUSG00000038388	Mpp6	0.0456533	2.86868
ENSMUSG00000046598	Bdh1	0.0456533	2.86871
ENSMUSG00000096852	Cyp2d12	0.0456533	2.86879
ENSMUSG00000015085	Entpd2	0.0456477	2.86987
ENSMUSG00000028943	Espn	0.0456286	2.87021
ENSMUSG00000007777	0610009B22Rik	0.0455763	2.87078
ENSMUSG00000085455	1810059H22Rik	0.0455763	2.87078
ENSMUSG00000082536	Gm13456	0.0455763	2.87111
ENSMUSG00000020089	Ppa1	0.045537	2.87169
ENSMUSG00000031590	Frg1	0.0454758	2.87232
ENSMUSG00000029063	Nadk	0.0454476	2.87294
ENSMUSG00000031289	Il13ra2	0.0452191	2.87516
ENSMUSG00000002718	Cse1l	0.0452191	2.87536
ENSMUSG00000030217	Art4	0.0452191	2.87563
ENSMUSG00000044303	Cdkn2a	0.0452191	2.87581
ENSMUSG00000049717	Lig4	0.0452191	2.87614
ENSMUSG00000028036	Ptgfr	0.0451863	2.87687
ENSMUSG00000046079	Lrrc8d	0.0451863	2.87693
ENSMUSG00000068083	Cyp2d40	0.04503	2.87881
ENSMUSG00000026873	Phf19	0.044934	2.87991
ENSMUSG00000038970	Lmtk2	0.0446859	2.88251
ENSMUSG00000066442	Mthfs	0.0446516	2.88296
ENSMUSG00000024512	Dynap	0.0446352	2.88329
ENSMUSG00000019027	Dnah1	0.044596	2.88378
ENSMUSG00000030587	2200002D01Rik	0.044596	2.8838
ENSMUSG00000028555	Ttc39a	0.044596	2.884
ENSMUSG00000030670	Cyp2r1	0.044596	2.88441
ENSMUSG00000058230	Grlf1	0.044596	2.88447
ENSMUSG00000015806	Qdpr	0.044596	2.88483
ENSMUSG00000049103	Ccr2	0.044596	2.88514
ENSMUSG00000081277	Gm15285	0.0445791	2.88561

ENSMUSG00000010760	Phlda2	0.0445226	2.88623
ENSMUSG00000071660	Ttc9c	0.0443799	2.88777
ENSMUSG00000038816	Ctnnal1	0.0441872	2.88968
ENSMUSG00000025403	Shmt2	0.0440229	2.89149
ENSMUSG00000051855	Mest	0.0438929	2.89285
ENSMUSG00000044122	Proca1	0.0435529	2.89573
ENSMUSG00000059022	Kcp	0.0430402	2.90031
ENSMUSG00000001911	Nfix	0.0430402	2.90038
ENSMUSG00000031494	Cd209a	0.0430402	2.90066
ENSMUSG00000015478	Rnf5	0.0429443	2.90165
ENSMUSG00000038591	Colec10	0.0427848	2.90335
ENSMUSG00000025165	Sectm1a	0.042735	2.90405
ENSMUSG00000022160	Mettl3	0.042735	2.90407
ENSMUSG00000048149	Gm12663	0.04272	2.90459
ENSMUSG00000035513	Ntng2	0.0424203	2.90701
ENSMUSG00000013593	Ndufs2	0.0424135	2.90728
ENSMUSG00000042385	Gzmk	0.0423475	2.90798
ENSMUSG00000000154	Slc22a18	0.0419261	2.91176
ENSMUSG00000081562	Gm11575	0.0416359	2.91415
ENSMUSG00000072664	Ugt3a1	0.0416359	2.91428
ENSMUSG00000038235	F11r	0.0414562	2.91593
ENSMUSG00000032369	Plscr1	0.0414452	2.91624
ENSMUSG00000024697	Gna14	0.0414436	2.91668
ENSMUSG00000038291	Snx25	0.0414436	2.91683
ENSMUSG00000056116	H2-T22	0.0413849	2.91756
ENSMUSG00000094530	Gm21399	0.0412749	2.91888
ENSMUSG00000028008	Asic5	0.041221	2.91967
ENSMUSG00000005373	Mlxipl	0.0410449	2.92163
ENSMUSG00000017400	Stac2	0.0409136	2.92288
ENSMUSG00000078970	Wdr92	0.0409136	2.92308
ENSMUSG00000083836	Ldha-ps2	0.0408933	2.92348
ENSMUSG00000034437	Gm9761	0.0404622	2.92743
ENSMUSG00000079057	Cyp4v3	0.0403565	2.92846
ENSMUSG00000002076	Hsf2bp	0.0403565	2.92871
ENSMUSG00000073481	Marc2	0.0399119	2.93345
ENSMUSG00000091898	Tnnc1	0.0398005	2.93454
ENSMUSG00000054408	Spcs3	0.0397424	2.93521
ENSMUSG00000041044	Lrit1	0.0393329	2.93997
ENSMUSG00000021228	Acot3	0.0392416	2.94097
ENSMUSG00000025198	Erlin1	0.0392127	2.94181
ENSMUSG00000073402	Gm8909	0.0391785	2.9423
ENSMUSG00000029699	Srcrb4d	0.0386212	2.94762
ENSMUSG00000041658	Rragb	0.038401	2.9505
ENSMUSG00000024844	Banf1	0.0383801	2.95089
ENSMUSG00000054942	Fam73a	0.0381704	2.95303
ENSMUSG00000031779	Ccl22	0.0379873	2.95474
ENSMUSG00000018846	Pank3	0.0379148	2.95555
ENSMUSG00000090623	Cfhr3	0.0376503	2.95816
ENSMUSG00000029188	Slc34a2	0.0376234	2.9586
ENSMUSG00000020423	Btg2	0.0373557	2.96125
ENSMUSG00000045502	Niacr1	0.0371246	2.96339
ENSMUSG00000038102	Trappc11	0.037017	2.9654
ENSMUSG00000075707	Dio3	0.0369463	2.96644
ENSMUSG00000050390	C77080	0.0369228	2.96686
ENSMUSG00000027514	Zbp1	0.0369228	2.96686
ENSMUSG00000021707	Dhfr	0.0369228	2.96702
ENSMUSG00000021048	Mthfd1	0.03686	2.96829
ENSMUSG00000025980	Hspd1	0.03686	2.96846
ENSMUSG00000057729	Prtn3	0.03686	2.96881
ENSMUSG00000079845	Xlr4a	0.036715	2.97085
ENSMUSG00000011305	Plin5	0.0366143	2.97193
ENSMUSG00000061104	Gm10094	0.0364427	2.97382
ENSMUSG00000085468	Gm15343	0.0364023	2.97461
ENSMUSG00000098872	RP23-103I12.13	0.036354	2.97525
ENSMUSG00000037278	Tmem97	0.0361272	2.97762
ENSMUSG00000049493	Pls1	0.0360796	2.97835
ENSMUSG00000045216	Hs6st1	0.0360796	2.97863
ENSMUSG00000054733	Msra	0.0360796	2.97875
ENSMUSG00000031373	Car5b	0.0359967	2.9801
ENSMUSG00000035561	Aldh1b1	0.0359561	2.98067
ENSMUSG00000075044	Slc22a29	0.0357886	2.98233
ENSMUSG00000023031	Cela1	0.0357886	2.98234
ENSMUSG00000069125	Rps24-ps2	0.0357886	2.98273

ENSMUSG00000023064	Sncg	0.0356785	2.98396
ENSMUSG00000093930	Hmgcs1	0.0355674	2.98515
ENSMUSG00000024805	Pcgf5	0.0355418	2.98559
ENSMUSG00000032458	Copb2	0.0353752	2.98773
ENSMUSG00000054293	A630033H20Rik	0.0353752	2.98794
ENSMUSG00000082330	Ldha-ps	0.0353392	2.98872
ENSMUSG00000032220	Myo1e	0.0353279	2.98974
ENSMUSG00000025492	Ifitm3	0.0349975	2.99284
ENSMUSG00000030663	1110004F10Rik	0.034905	2.99434
ENSMUSG00000078853	Igtp	0.0348928	2.99468
ENSMUSG00000042289	Hsd3b7	0.0348329	2.99566
ENSMUSG00000061544	Zfp229	0.0347935	2.99641
ENSMUSG00000019947	Arid5b	0.0347878	2.99675
ENSMUSG00000024907	Gal	0.0347699	2.99795
ENSMUSG00000044768	D1Ertd622e	0.0344908	3.00076
ENSMUSG00000020514	Mrpl22	0.0339345	3.00664
ENSMUSG00000048351	Selrc1	0.0339345	3.00678
ENSMUSG00000029126	Nsg1	0.0338084	3.00832
ENSMUSG00000086748	Gm13261	0.0338084	3.00837
ENSMUSG00000021868	Ppif	0.0338082	3.00918
ENSMUSG00000028967	Errfi1	0.0338082	3.00933
ENSMUSG00000028792	Ak2	0.0335586	3.01189
ENSMUSG00000090925	1810064F22Rik	0.0334764	3.01287
ENSMUSG00000050164	Mchr1	0.0333932	3.0141
ENSMUSG00000072582	Ptrh2	0.0333106	3.01508
ENSMUSG00000046711	Hmga1	0.0331805	3.01651
ENSMUSG00000015357	Clpx	0.0331493	3.01702
ENSMUSG00000048040	Arxes2	0.0331493	3.01705
ENSMUSG00000029086	Prom1	0.0328992	3.02003
ENSMUSG00000040714	Klc3	0.0327766	3.02186
ENSMUSG00000038065	2410066E13Rik	0.0327579	3.02227
ENSMUSG00000039682	Lap3	0.0326762	3.02326
ENSMUSG00000013663	Pten	0.0326598	3.02365
ENSMUSG00000031596	Slc7a2	0.0325488	3.02539
ENSMUSG00000036751	Cox6b1	0.0324404	3.02664
ENSMUSG00000022236	Ropn1l	0.0324404	3.02683
ENSMUSG00000048924	Ccdc125	0.0323721	3.02775
ENSMUSG00000020321	Mdh1	0.0322423	3.0292
ENSMUSG00000034951	Cog7	0.0320318	3.03212
ENSMUSG00000020911	Krt19	0.0320149	3.03252
ENSMUSG00000066513	Klk1b4	0.0319695	3.03366
ENSMUSG00000021786	Oxsm	0.0318726	3.03519
ENSMUSG00000018574	Acadvl	0.0315532	3.03893
ENSMUSG00000063856	Gpx1	0.0315532	3.0391
ENSMUSG00000053168	9030619P08Rik	0.031297	3.04198
ENSMUSG00000063730	Hsd3b2	0.0311492	3.04412
ENSMUSG00000005566	Trim28	0.030966	3.04614
ENSMUSG00000001674	Ddx18	0.0308614	3.04788
ENSMUSG00000036136	Fam110c	0.0308112	3.0486
ENSMUSG00000040034	Nup43	0.0307961	3.04899
ENSMUSG00000035351	Nup37	0.0304829	3.05309
ENSMUSG00000017801	Mlx	0.0304822	3.05351
ENSMUSG00000041444	Arhgap32	0.0302753	3.0558
ENSMUSG00000015247	Nipsnap3b	0.0302018	3.05677
ENSMUSG00000001672	Marveld3	0.0301857	3.05723
ENSMUSG00000028696	Ipp	0.0301791	3.05772
ENSMUSG00000032718	Mansc1	0.0300899	3.05885
ENSMUSG00000032735	Ablim3	0.0300899	3.05892
ENSMUSG00000022762	Ncam2	0.0300899	3.05941
ENSMUSG00000003585	Sec14l2	0.0299561	3.0617
ENSMUSG00000028780	Sema3c	0.0298713	3.06297
ENSMUSG00000020733	Slc9a3r1	0.0298274	3.06366
ENSMUSG00000025545	Clybl	0.0298274	3.06369
ENSMUSG00000086436	Gm13690	0.02941	3.0686
ENSMUSG00000027375	Mal	0.0292707	3.07075
ENSMUSG00000056498	Tmem154	0.0292707	3.07092
ENSMUSG00000036216	Leap2	0.0290944	3.07305
ENSMUSG00000036078	Sigmar1	0.0289179	3.07511
ENSMUSG00000015023	Ddx19a	0.0288969	3.07557
ENSMUSG00000098747	RP23-341H6.1	0.0288718	3.07607
ENSMUSG00000043969	Emx2	0.0288718	3.07621
ENSMUSG00000097000	Gm17435	0.0288706	3.07657
ENSMUSG00000086868	Gm15883	0.0287212	3.07861

ENSMUSG00000078532	Nkain1	0.0286912	3.07917
ENSMUSG00000032468	Armc8	0.0286408	3.07994
ENSMUSG00000097732	D930048G16Rik	0.0286408	3.08025
ENSMUSG00000048764	Tmprss11f	0.0284418	3.083
ENSMUSG00000023186	Vwa5a	0.0282916	3.08482
ENSMUSG00000028965	Tnfrsf9	0.0280942	3.08764
ENSMUSG00000028405	Aco1	0.0276713	3.09239
ENSMUSG00000020171	Yeats4	0.0275404	3.09406
ENSMUSG00000022244	Amacr	0.0275124	3.09534
ENSMUSG00000042462	Dctpp1	0.0274616	3.09638
ENSMUSG00000022887	Masp1	0.0274163	3.09712
ENSMUSG00000037762	Slc16a9	0.0274065	3.09748
ENSMUSG00000024151	Msh2	0.0272755	3.09939
ENSMUSG00000000934	Top1mt	0.0271114	3.10143
ENSMUSG00000022686	B3gnt5	0.0270206	3.10267
ENSMUSG00000036655	Colec11	0.0266866	3.10685
ENSMUSG00000033898	Cfhr2	0.0266153	3.10839
ENSMUSG00000034855	Cxcl10	0.0264614	3.11035
ENSMUSG00000017390	Aldoc	0.0264394	3.11084
ENSMUSG00000062284	Gm6030	0.026153	3.11456
ENSMUSG00000022016	Akap11	0.0259728	3.1171
ENSMUSG00000031960	Aars	0.0259612	3.11748
ENSMUSG00000030744	Rps3	0.0258496	3.11925
ENSMUSG00000022351	Sqle	0.0252991	3.1276
ENSMUSG00000030469	Zfp719	0.0252294	3.12916
ENSMUSG00000073234	Gm8773	0.0250817	3.13114
ENSMUSG00000008373	Prpf31	0.0250714	3.13151
ENSMUSG00000022193	Psmb5	0.0250023	3.13283
ENSMUSG00000039110	Mycbpap	0.0249348	3.13413
ENSMUSG00000020850	Prpf8	0.0248084	3.13587
ENSMUSG00000027227	Sord	0.0247119	3.13727
ENSMUSG00000040859	Bsdc1	0.0246698	3.13819
ENSMUSG00000022041	Chrna2	0.0246122	3.13922
ENSMUSG00000024683	Mrpl16	0.024398	3.14203
ENSMUSG00000054793	Cadm4	0.024398	3.14225
ENSMUSG00000078153	Psme2b	0.0238055	3.14998
ENSMUSG00000038541	Srd5a2	0.0237929	3.15039
ENSMUSG00000003429	Rps11	0.0237247	3.152
ENSMUSG00000046027	Stard5	0.0237247	3.15207
ENSMUSG00000027956	Tmem144	0.0236059	3.15433
ENSMUSG00000026390	Marco	0.0236059	3.15463
ENSMUSG00000086010	Gm15318	0.023561	3.15555
ENSMUSG00000096062	Pik3c2g	0.0235383	3.15609
ENSMUSG00000041625	Ggact	0.0234967	3.15686
ENSMUSG00000021367	Edn1	0.0234706	3.15767
ENSMUSG00000057068	Fam47e	0.0234706	3.15774
ENSMUSG00000028393	Alad	0.02345	3.15847
ENSMUSG00000013367	Iglon5	0.0234331	3.15894
ENSMUSG00000035783	Acta2	0.0231589	3.16314
ENSMUSG00000027981	Rnpc3	0.022911	3.16704
ENSMUSG00000098234	Snhg6	0.022911	3.16723
ENSMUSG00000083813	Gm15502	0.0228825	3.16818
ENSMUSG00000041272	Tox	0.0228593	3.16873
ENSMUSG00000024601	Isoc1	0.0228584	3.169
ENSMUSG00000044788	Fads6	0.0228584	3.16905
ENSMUSG00000009654	Oit3	0.0228584	3.16908
ENSMUSG00000041481	Serpina3g	0.0224134	3.17653
ENSMUSG00000019989	Enpp3	0.0221617	3.18006
ENSMUSG00000022425	Enpp2	0.022059	3.18193
ENSMUSG00000031349	Nsdhl	0.0219247	3.18396
ENSMUSG00000024981	Acsl5	0.0219247	3.18411
ENSMUSG00000010122	Slc47a1	0.021826	3.18605
ENSMUSG00000024875	Yif1a	0.0217901	3.18679
ENSMUSG00000034321	Exosc1	0.0217163	3.18829
ENSMUSG00000090145	Ugt1a6b	0.0216169	3.18988
ENSMUSG00000000168	Dlat	0.0216169	3.18996
ENSMUSG00000062432	Cyp26c1	0.021602	3.1906
ENSMUSG00000025059	Gyk	0.021602	3.19072
ENSMUSG00000039202	Abhd2	0.021517	3.19227
ENSMUSG00000097673	Gm26608	0.0214843	3.19323
ENSMUSG00000038764	Ptpn3	0.0213877	3.1948
ENSMUSG00000052456	Asna1	0.0211881	3.19777
ENSMUSG00000059070	Rpl18	0.0211751	3.19821

ENSMUSG00000018171	Vmp1	0.0211485	3.1991
ENSMUSG00000062181	Ces3b	0.0210564	3.20062
ENSMUSG00000067608	Pcna-ps2	0.0210564	3.20082
ENSMUSG00000023921	Mut	0.0210564	3.20095
ENSMUSG00000026123	Plekhb2	0.0210183	3.20246
ENSMUSG00000075517	Cyp2d37-ps	0.0209318	3.20392
ENSMUSG00000030214	Plbd1	0.0208989	3.20495
ENSMUSG00000098168	D630036G22Rik	0.0208434	3.2062
ENSMUSG00000021884	Hacl1	0.0207715	3.20746
ENSMUSG00000028609	Magoh	0.0206732	3.20909
ENSMUSG00000046826	Fam187b	0.020641	3.21007
ENSMUSG00000044923	Olfr1030	0.0206072	3.21081
ENSMUSG00000094664	Rpl35a-ps6	0.0205727	3.21182
ENSMUSG00000036813	Entpd8	0.0205727	3.21194
ENSMUSG00000033885	Pxk	0.0205727	3.21217
ENSMUSG00000026730	Pter	0.0204734	3.21482
ENSMUSG00000035078	Mtmr9	0.0202264	3.21857
ENSMUSG00000032014	Oaf	0.0199315	3.22332
ENSMUSG00000035517	Tdrd7	0.0197966	3.2258
ENSMUSG00000003411	Rab3b	0.0197658	3.22652
ENSMUSG00000078954	Arhgap8	0.0197658	3.22666
ENSMUSG00000052221	Ppp1r36	0.0197441	3.22737
ENSMUSG00000024165	Hn1l	0.0197163	3.22805
ENSMUSG00000022040	Ephx2	0.0196808	3.2291
ENSMUSG00000020361	Hspa4	0.0196262	3.23017
ENSMUSG00000040498	Igsf23	0.0195636	3.23135
ENSMUSG00000021678	F2rl1	0.0195474	3.23186
ENSMUSG00000029664	Tfpi2	0.0195474	3.232
ENSMUSG00000035105	Egln3	0.0195474	3.23223
ENSMUSG00000064325	Hhip	0.0194835	3.23373
ENSMUSG00000045991	Onecut2	0.0194835	3.2338
ENSMUSG00000020553	Pctp	0.0194423	3.23503
ENSMUSG00000001440	Kpnb1	0.0193603	3.23651
ENSMUSG00000053182	Gm609	0.0193126	3.23748
ENSMUSG00000038259	Gdf5	0.0190935	3.2421
ENSMUSG00000032883	Acsl3	0.019045	3.2431
ENSMUSG00000024942	Capn1	0.019045	3.24312
ENSMUSG00000031016	Wee1	0.019045	3.2433
ENSMUSG00000022574	Naprt1	0.0189418	3.24575
ENSMUSG00000029822	Osbpl3	0.0189013	3.24663
ENSMUSG00000027171	Prrg4	0.0189013	3.24686
ENSMUSG00000053303	Slc22a26	0.0187351	3.25025
ENSMUSG00000022408	Fam83f	0.0186693	3.25207
ENSMUSG00000025465	Echs1	0.0186574	3.25253
ENSMUSG00000001349	Cnn1	0.0186503	3.25319
ENSMUSG00000079362	Gbp6	0.0186166	3.25399
ENSMUSG00000066232	Ipo7	0.0185796	3.25483
ENSMUSG00000061559	Wdr61	0.0185773	3.25514
ENSMUSG00000028024	Enpep	0.0185773	3.25532
ENSMUSG00000026172	Bcs1l	0.018551	3.2561
ENSMUSG00000005917	Otx1	0.0185463	3.25673
ENSMUSG00000034785	Dio1	0.0185463	3.25688
ENSMUSG00000090231	Cfb	0.018542	3.25735
ENSMUSG00000097482	Gm17634	0.0184557	3.25924
ENSMUSG00000042476	Abcb4	0.0183917	3.2605
ENSMUSG00000002346	Slc25a42	0.0183247	3.2621
ENSMUSG00000089726	Mir17hg	0.0183247	3.26228
ENSMUSG00000070407	Hs3st3b1	0.0183054	3.26296
ENSMUSG00000011034	Slc5a1	0.018235	3.26436
ENSMUSG00000033498	Strc	0.0180699	3.26775
ENSMUSG00000038768	9130409I23Rik	0.0180336	3.2686
ENSMUSG00000028354	Fmn2	0.0180298	3.26894
ENSMUSG00000085711	Gm15163	0.0180298	3.26905
ENSMUSG00000017002	Slpi	0.0180298	3.26924
ENSMUSG00000033216	Eefsec	0.0178834	3.27238
ENSMUSG00000034274	Thoc5	0.0178834	3.27249
ENSMUSG00000006818	Sod2	0.0177425	3.27546
ENSMUSG00000037600	1810019J16Rik	0.0174445	3.28081
ENSMUSG00000034308	Sdr42e1	0.017393	3.28193
ENSMUSG00000073491	Pydc4	0.017306	3.28363
ENSMUSG00000058076	Sdhc	0.017306	3.28393
ENSMUSG00000062209	Erbb4	0.0172536	3.28562
ENSMUSG00000016520	Lnx2	0.0172315	3.28627

ENSMUSG00000078867	Gm14418	0.0171822	3.28736
ENSMUSG00000018340	Anxa6	0.0171333	3.28845
ENSMUSG00000025503	Taldo1	0.0170396	3.29056
ENSMUSG00000050103	Agmo	0.0169942	3.2916
ENSMUSG00000020526	Znhit3	0.0168636	3.29405
ENSMUSG00000063802	Hspbp1	0.0168005	3.29539
ENSMUSG00000027762	Sucnr1	0.0167972	3.29574
ENSMUSG00000081988	Gm15746	0.0166471	3.29854
ENSMUSG00000071014	Ndufb6	0.0166118	3.29943
ENSMUSG00000031778	Cx3cl1	0.0165239	3.3012
ENSMUSG00000043618	Eif5al3-ps	0.0163291	3.30491
ENSMUSG00000045691	Thtpa	0.0163154	3.30596
ENSMUSG00000005469	Prkaca	0.0162606	3.30801
ENSMUSG00000085492	Trmt61b	0.0162405	3.30865
ENSMUSG00000021908	Gm6768	0.0162405	3.30905
ENSMUSG00000027457	Snph	0.016223	3.30988
ENSMUSG00000054545	Ugt1a6a	0.016223	3.31041
ENSMUSG00000068039	Tcp1	0.0161806	3.31172
ENSMUSG00000038217	Tlcd2	0.016162	3.31233
ENSMUSG00000021944	Gata4	0.0161125	3.31357
ENSMUSG00000028409	Smu1	0.0161125	3.31367
ENSMUSG00000031672	Got2	0.0160397	3.31562
ENSMUSG00000025780	Itih5	0.0159357	3.31773
ENSMUSG00000036377	C530008M17Rik	0.0157643	3.32193
ENSMUSG00000031549	Ido2	0.015756	3.32237
ENSMUSG00000066842	Hmcn1	0.015699	3.32367
ENSMUSG00000021996	Esd	0.0156513	3.32481
ENSMUSG00000039953	Clstn1	0.015318	3.3311
ENSMUSG00000019935	Slc17a8	0.015215	3.33387
ENSMUSG00000054619	Mettl7a1	0.0152011	3.33442
ENSMUSG00000097928	Gm26578	0.0150412	3.33883
ENSMUSG00000056973	Ces1d	0.0149781	3.34051
ENSMUSG00000090610	Gm3571	0.0149088	3.34248
ENSMUSG00000028327	1300002K09Rik	0.0149088	3.3425
ENSMUSG00000081434	Gm14165	0.0149088	3.3428
ENSMUSG00000037742	Eef1a1	0.0149088	3.34334
ENSMUSG00000097310	A930038B10Rik	0.014825	3.34553
ENSMUSG00000032050	Rdx	0.0147188	3.34917
ENSMUSG00000036291	Ap5m1	0.0146866	3.35023
ENSMUSG00000083626	Gm4459	0.0146826	3.35061
ENSMUSG00000028088	Fmo5	0.0146822	3.35091
ENSMUSG00000026255	Efhd1	0.0146568	3.3523
ENSMUSG00000054676	1600014C10Rik	0.0146134	3.35342
ENSMUSG00000026348	Acmsd	0.0145856	3.35425
ENSMUSG00000027831	Veph1	0.0145141	3.35591
ENSMUSG00000034783	Cd207	0.0145141	3.35609
ENSMUSG00000030965	Fam175b	0.0143253	3.36044
ENSMUSG00000028158	Mttp	0.0142481	3.36254
ENSMUSG00000074639	BC089597	0.0142481	3.3628
ENSMUSG00000029153	Ociad2	0.0142481	3.36299
ENSMUSG00000025078	Nhlrc2	0.0142481	3.36344
ENSMUSG00000097754	Gm26687	0.0140756	3.36865
ENSMUSG00000035498	Cdcp1	0.0138279	3.37397
ENSMUSG00000044018	Mrpl50	0.0137378	3.37657
ENSMUSG00000021187	Tc2n	0.0137096	3.37744
ENSMUSG00000048988	Elfn1	0.0136943	3.37806
ENSMUSG00000046402	Rbp1	0.0136733	3.37909
ENSMUSG00000029273	Sult1d1	0.0136733	3.37939
ENSMUSG00000048489	8430408G22Rik	0.0136733	3.37961
ENSMUSG00000052942	Glis3	0.0136144	3.38167
ENSMUSG00000056569	Mpz	0.0134313	3.38585
ENSMUSG00000037071	Scd1	0.0134313	3.38589
ENSMUSG00000086494	2210417A02Rik	0.0134178	3.38675
ENSMUSG00000027035	Cers6	0.0134003	3.38742
ENSMUSG00000019256	Ahr	0.0133692	3.38846
ENSMUSG00000030200	Bcl2l14	0.0133692	3.38892
ENSMUSG00000053897	Slc39a8	0.0133692	3.38914
ENSMUSG00000051397	Tacstd2	0.0133692	3.38953
ENSMUSG00000031342	Gpm6b	0.0133692	3.3896
ENSMUSG00000038462	Uqcrfs1	0.0133692	3.38985
ENSMUSG00000041930	Fam222a	0.0132971	3.39235
ENSMUSG00000029663	Gngt1	0.0132422	3.39411
ENSMUSG00000021213	Akr1c13	0.0132308	3.39466

ENSMUSG00000032193	Ldlr	0.0132308	3.39488
ENSMUSG00000027356	Fermt1	0.0130106	3.4002
ENSMUSG00000041939	Mvk	0.0128187	3.40489
ENSMUSG00000029322	Plac8	0.0127672	3.40631
ENSMUSG00000040537	Adam22	0.0126419	3.40931
ENSMUSG00000031700	Gpt2	0.0126419	3.40946
ENSMUSG00000037185	Krt80	0.0125956	3.41095
ENSMUSG00000020037	Rfx4	0.012491	3.41386
ENSMUSG00000086628	Gm16157	0.012491	3.41401
ENSMUSG00000024997	Prdx3	0.012491	3.41415
ENSMUSG00000020456	Ogdh	0.012364	3.41759
ENSMUSG00000060227	Casc4	0.0123341	3.41889
ENSMUSG00000069744	Psmb3	0.0123256	3.41939
ENSMUSG00000026187	Xrcc5	0.0123053	3.42016
ENSMUSG00000040550	Otud6b	0.0123053	3.42033
ENSMUSG00000015966	Il17rb	0.0122754	3.42146
ENSMUSG00000096035	Gm1045	0.0122754	3.42152
ENSMUSG00000078763	Slfn1	0.0122754	3.42168
ENSMUSG00000079045	Gm17566	0.0122754	3.42188
ENSMUSG00000068964	Gm6361	0.0122754	3.42259
ENSMUSG00000055900	Tmem69	0.0121432	3.42633
ENSMUSG00000078862	Gm14326	0.0120611	3.42849
ENSMUSG00000043681	Fam25c	0.0120553	3.42894
ENSMUSG00000041798	Gck	0.0119734	3.43112
ENSMUSG00000026109	Tmeff2	0.0119734	3.43134
ENSMUSG00000032381	Fam96a	0.0119734	3.43145
ENSMUSG00000023885	Thbs2	0.0119596	3.4324
ENSMUSG00000026616	Cr2	0.0119414	3.43314
ENSMUSG00000046314	Stxbp6	0.0119234	3.43387
ENSMUSG00000039735	Fnbp1l	0.0118731	3.43534
ENSMUSG00000055629	B4galnt4	0.0118683	3.43577
ENSMUSG00000027208	Fgf7	0.0117753	3.43855
ENSMUSG00000023830	Igf2r	0.0116352	3.44277
ENSMUSG00000030934	Oat	0.0116065	3.44376
ENSMUSG00000046380	Jrk	0.011545	3.44553
ENSMUSG00000055254	Ntrk2	0.011545	3.44572
ENSMUSG00000003949	Hlf	0.0115264	3.44662
ENSMUSG00000032047	Acat1	0.0114718	3.44823
ENSMUSG00000026229	Psmd1	0.0114465	3.44948
ENSMUSG00000023122	Sult1c2	0.0114393	3.44998
ENSMUSG00000045763	Basp1	0.0112616	3.45453
ENSMUSG00000059873	Olfr1029	0.0112616	3.4547
ENSMUSG00000068762	Gstm6	0.0112343	3.45585
ENSMUSG00000059743	Fdps	0.0111555	3.4584
ENSMUSG00000027536	Chmp4c	0.0110932	3.46024
ENSMUSG00000040706	Agmat	0.0110861	3.46074
ENSMUSG00000048905	4930539E08Rik	0.0110268	3.46252
ENSMUSG00000005148	Klf5	0.0110268	3.46269
ENSMUSG00000028003	Lrat	0.0110268	3.46271
ENSMUSG00000020123	Avpr1a	0.0110129	3.46418
ENSMUSG00000096546	Smlr1	0.0108815	3.46774
ENSMUSG00000040658	Dnph1	0.0108625	3.46855
ENSMUSG00000026581	Sell	0.0104781	3.47921
ENSMUSG00000040525	Cblc	0.0103857	3.48225
ENSMUSG00000022763	Aifm3	0.0103062	3.48531
ENSMUSG00000045094	Arhgef37	0.0102367	3.48779
ENSMUSG00000022587	Ly6e	0.0102358	3.48815
ENSMUSG00000055760	Gemin6	0.0102358	3.4882
ENSMUSG00000075297	H60b	0.0101874	3.49009
ENSMUSG00000044033	Ccdc141	0.0101582	3.4912
ENSMUSG00000024228	Nudt12	0.0101029	3.49299
ENSMUSG00000032487	Ptgs2	0.00998083	3.49657
ENSMUSG00000072115	Ang	0.00996473	3.49792
ENSMUSG00000031604	Sc4mol	0.0098504	3.50143
ENSMUSG00000032314	Etfa	0.00974683	3.5056
ENSMUSG00000072625	Gdf2	0.0097078	3.50701
ENSMUSG00000024158	Hagh	0.00967107	3.50836
ENSMUSG00000067212	H2-T23	0.00961814	3.51034
ENSMUSG00000054702	Ap1s3	0.00961814	3.51042
ENSMUSG00000000440	Pparg	0.00953458	3.51385
ENSMUSG00000027531	Impa1	0.00953458	3.51417
ENSMUSG00000031343	Gabra3	0.00952139	3.5149
ENSMUSG00000003464	Pex19	0.00945992	3.51696

ENSMUSG00000098050	Gm5345	0.0094388	3.5179
ENSMUSG00000024109	Nrxn1	0.0094388	3.5182
ENSMUSG00000020900	Myh10	0.0094388	3.51825
ENSMUSG00000021771	Vdac2	0.00932804	3.5231
ENSMUSG00000002981	Clptm1	0.00922693	3.52633
ENSMUSG00000026356	Dars	0.00918715	3.52817
ENSMUSG00000025260	Hsd17b10	0.00915662	3.52974
ENSMUSG00000022025	Lect1	0.00908681	3.53212
ENSMUSG00000010601	Apol7a	0.00908681	3.53234
ENSMUSG00000040963	Asgr2	0.00905002	3.53389
ENSMUSG00000024866	Acy3	0.00894595	3.53799
ENSMUSG00000027597	Ahcy	0.00894232	3.53845
ENSMUSG00000024074	Crim1	0.00889317	3.54025
ENSMUSG00000074472	Zfp872	0.00885165	3.54184
ENSMUSG00000020372	Gnb2l1	0.00877884	3.54472
ENSMUSG00000052369	Tmem106c	0.00877881	3.54507
ENSMUSG00000044968	Napepld	0.00869662	3.548
ENSMUSG00000030031	Kbtbd8	0.00868826	3.54886
ENSMUSG00000018286	Psmb6	0.00866907	3.55015
ENSMUSG00000026893	Gca	0.00854751	3.55457
ENSMUSG00000069825	Spata22	0.0084913	3.55701
ENSMUSG00000040891	Foxa3	0.00844563	3.55889
ENSMUSG00000083716	Gm13436	0.00839806	3.56098
ENSMUSG00000059742	Kcnh7	0.0083438	3.5634
ENSMUSG00000086593	Gm16548	0.00828309	3.56567
ENSMUSG00000092545	Gm20319	0.00820581	3.56997
ENSMUSG00000032500	Dclk3	0.00816632	3.5719
ENSMUSG00000082209	Eif4a-ps4	0.00816305	3.57237
ENSMUSG00000039238	Zfp750	0.00812168	3.57442
ENSMUSG00000074637	Sox2	0.00810366	3.57536
ENSMUSG00000030681	Mvp	0.00807817	3.57655
ENSMUSG00000078920	Ifi47	0.00805151	3.57777
ENSMUSG00000053030	Spink2	0.00804529	3.57834
ENSMUSG00000031958	Ldhd	0.0079656	3.5813
ENSMUSG00000039457	Ppl	0.00782477	3.58632
ENSMUSG00000053886	Sh2d4a	0.00782445	3.5867
ENSMUSG00000081824	BC002163	0.00770735	3.59209
ENSMUSG00000030750	Nsmce1	0.00763887	3.59514
ENSMUSG00000006179	Prss16	0.00761384	3.59636
ENSMUSG00000074829	2010315B03Rik	0.00760545	3.59702
ENSMUSG00000044117	2900011O08Rik	0.0075852	3.59808
ENSMUSG00000043613	Mmp3	0.00757707	3.59872
ENSMUSG00000019883	Echdc1	0.00754677	3.60027
ENSMUSG00000054065	Pkp3	0.00754677	3.6005
ENSMUSG00000094910	D430019H16Rik	0.00754677	3.60076
ENSMUSG00000005320	Fgfr4	0.00732442	3.61012
ENSMUSG00000015652	Steap1	0.00730439	3.6112
ENSMUSG00000075225	Ccdc162	0.00719933	3.6157
ENSMUSG00000070661	Rnf186	0.00719933	3.61605
ENSMUSG00000079852	Klra4	0.00715205	3.61852
ENSMUSG00000020431	Adcy1	0.00710261	3.62069
ENSMUSG00000025533	Asl	0.00709926	3.62118
ENSMUSG00000031274	Col4a5	0.00709165	3.62183
ENSMUSG00000026043	Col3a1	0.00704388	3.62396
ENSMUSG00000024982	Zdhhc6	0.00695947	3.62745
ENSMUSG00000030521	Mphosph10	0.00694803	3.62862
ENSMUSG00000059434	Gckr	0.00690604	3.63092
ENSMUSG00000064637	Snora20	0.0068654	3.63284
ENSMUSG00000024694	Keg1	0.00677024	3.63681
ENSMUSG00000026986	Hnmt	0.00674667	3.63809
ENSMUSG00000028712	Cyp4a31	0.00664862	3.64224
ENSMUSG00000031661	Nkd1	0.00664862	3.64238
ENSMUSG00000023073	Slc10a2	0.00656807	3.64689
ENSMUSG00000065952	C330021F23Rik	0.00655313	3.64785
ENSMUSG00000061838	Suclg2	0.00642652	3.65401
ENSMUSG00000025911	Adhfe1	0.00615148	3.66727
ENSMUSG00000028347	Tmeff1	0.00603663	3.67271
ENSMUSG00000033610	Pank1	0.00601435	3.67403
ENSMUSG00000035473	Galm	0.00601435	3.67429
ENSMUSG00000036292	Gramd1c	0.00598538	3.67604
ENSMUSG00000039062	Anpep	0.00598538	3.67641
ENSMUSG00000097426	Gm8941	0.00598538	3.67717
ENSMUSG00000010064	Slc38a3	0.00598538	3.67749

ENSMUSG00000031645	F11	0.00596828	3.67909
ENSMUSG00000040434	Gyltl1b	0.00593795	3.68077
ENSMUSG00000010154	Spire2	0.00592999	3.6815
ENSMUSG00000055653	Gpc3	0.00591075	3.68311
ENSMUSG00000026343	Gpr39	0.00589706	3.68448
ENSMUSG00000053846	Lipg	0.00580411	3.68892
ENSMUSG00000026768	Itga8	0.00576892	3.69086
ENSMUSG00000022865	Cxadr	0.00576892	3.691
ENSMUSG00000046811	Gltpd2	0.00575853	3.6921
ENSMUSG00000020875	Hoxb9	0.00575853	3.69225
ENSMUSG00000028051	Hcn3	0.00575853	3.6927
ENSMUSG00000026234	Ncl	0.00575853	3.6929
ENSMUSG00000048376	F2r	0.00572613	3.69551
ENSMUSG00000091618	H60c	0.00569633	3.69723
ENSMUSG00000066319	Rtp3	0.00562988	3.701
ENSMUSG00000042213	Zfand4	0.00554014	3.70558
ENSMUSG00000032387	Rbpms2	0.00548338	3.70888
ENSMUSG00000003526	Prodh	0.00544435	3.71108
ENSMUSG00000034371	Dak	0.00544435	3.71148
ENSMUSG00000026473	Glul	0.00542226	3.71371
ENSMUSG00000023391	Dlx2	0.00539418	3.71543
ENSMUSG00000028837	Psmb2	0.00537598	3.71668
ENSMUSG00000052562	Slc22a30	0.005279	3.72168
ENSMUSG00000070985	Acnat1	0.005279	3.72171
ENSMUSG00000026542	Apcs	0.005279	3.72201
ENSMUSG00000020072	Pbld1	0.00524188	3.72467
ENSMUSG00000030029	Lrig1	0.00524188	3.72495
ENSMUSG00000034917	Tjp3	0.00524188	3.72524
ENSMUSG00000035459	Stab2	0.00521501	3.72718
ENSMUSG00000038984	Tspyl5	0.0051814	3.72922
ENSMUSG00000018848	Rars	0.0051599	3.73067
ENSMUSG00000037541	Shank2	0.0051599	3.73096
ENSMUSG00000056966	Gjc3	0.00513163	3.73287
ENSMUSG00000085172	Gm6542	0.00511541	3.73407
ENSMUSG00000028357	Kif12	0.00511541	3.73448
ENSMUSG00000045954	Sdpr	0.00506146	3.73797
ENSMUSG00000071177	Serpina1d	0.00506146	3.73799
ENSMUSG00000028434	Epb4.1l4b	0.00487202	3.74837
ENSMUSG00000071633	Gm4952	0.00487202	3.74847
ENSMUSG00000032481	Smarcc1	0.00487202	3.74898
ENSMUSG00000048482	Bdnf	0.00485428	3.75051
ENSMUSG00000097906	Gm9625	0.00481872	3.75318
ENSMUSG00000046794	Ppp1r3b	0.00479263	3.75537
ENSMUSG00000038732	Mboat1	0.00471797	3.76096
ENSMUSG00000058355	Abce1	0.00468771	3.76298
ENSMUSG00000032204	Aqp9	0.00468771	3.76328
ENSMUSG00000089768	Tmsb15b1	0.00468673	3.76387
ENSMUSG00000029597	Sds	0.00467302	3.76502
ENSMUSG00000051177	Plcb1	0.00465616	3.76634
ENSMUSG00000039004	Bmp6	0.00465445	3.76685
ENSMUSG00000051652	Lrrc3	0.00464624	3.76856
ENSMUSG00000042082	Arsb	0.00450352	3.77718
ENSMUSG00000026574	Dpt	0.00450352	3.77727
ENSMUSG00000037780	Mbl1	0.00446595	3.78011
ENSMUSG00000020017	Hal	0.00445985	3.78113
ENSMUSG00000017978	Cadps2	0.0043352	3.79005
ENSMUSG00000027261	Hao1	0.00421804	3.79856
ENSMUSG00000028603	Scp2	0.00420474	3.80021
ENSMUSG00000020849	Ywhae	0.00420474	3.80055
ENSMUSG00000031533	Mrps31	0.00407999	3.80852
ENSMUSG00000093916	Gm379	0.00393862	3.81767
ENSMUSG00000058454	Dhcr7	0.00384172	3.82467
ENSMUSG00000037405	Icam1	0.00377993	3.82953
ENSMUSG00000003477	Inmt	0.00377993	3.82961
ENSMUSG00000030541	Idh2	0.00370151	3.83555
ENSMUSG00000039470	Zdhhc2	0.00365033	3.8394
ENSMUSG00000042797	Aqp11	0.00365033	3.83976
ENSMUSG00000041261	Car8	0.0036145	3.84313
ENSMUSG00000030834	Abcc6	0.00358816	3.84536
ENSMUSG00000028536	2610528J11Rik	0.00357526	3.84704
ENSMUSG00000055546	Timd4	0.00357388	3.84765
ENSMUSG00000024892	Pcx	0.00357217	3.8482
ENSMUSG00000035878	Hykk	0.00357217	3.8486

ENSMUSG00000034456	Uroc1	0.00351657	3.85337
ENSMUSG00000074227	Spint2	0.00348306	3.85615
ENSMUSG00000026701	Prdx6	0.00345771	3.85838
ENSMUSG00000092056	Gm7289	0.00340733	3.86241
ENSMUSG00000069170	Gpr98	0.00340069	3.86333
ENSMUSG00000019806	Aig1	0.00340069	3.86376
ENSMUSG00000024589	Nedd4l	0.00338553	3.8653
ENSMUSG00000025396	Hsd17b6	0.00331654	3.87077
ENSMUSG00000031984	2810004N23Rik	0.003142	3.88482
ENSMUSG00000021062	Rab15	0.003085	3.88971
ENSMUSG00000041782	Lad1	0.003085	3.89011
ENSMUSG00000045394	Epcam	0.0030738	3.89148
ENSMUSG00000085995	Gm2788	0.00303305	3.89516
ENSMUSG00000021748	Pdhb	0.00303305	3.89596
ENSMUSG00000005677	Nr1i3	0.00303299	3.89696
ENSMUSG00000002984	Tomm40	0.00295493	3.90644
ENSMUSG00000029269	Sult1b1	0.00292072	3.91017
ENSMUSG00000001864	Aif1l	0.00291315	3.91125
ENSMUSG00000073000	Gm10451	0.00289245	3.91343
ENSMUSG00000025194	Abcc2	0.00284748	3.91905
ENSMUSG00000034118	Tpst1	0.00284748	3.91921
ENSMUSG00000032808	Cyp2c38	0.00280474	3.92408
ENSMUSG00000058921	Slc10a5	0.00278337	3.92707
ENSMUSG00000045252	Zfp574	0.00277566	3.92845
ENSMUSG00000066072	Cyp4a10	0.00277457	3.92901
ENSMUSG00000022534	Mefv	0.00276261	3.93051
ENSMUSG00000069324	Gm5096	0.00276261	3.93066
ENSMUSG00000040017	Saa4	0.00276261	3.93076
ENSMUSG00000028864	Hgf	0.0027527	3.93278
ENSMUSG00000042248	Cyp2c37	0.00274414	3.934
ENSMUSG00000039529	Atp8b1	0.00274414	3.93419
ENSMUSG00000029162	Khk	0.00274414	3.93439
ENSMUSG00000052117	D630039A03Rik	0.00269003	3.94067
ENSMUSG00000035875	AI182371	0.00264314	3.94584
ENSMUSG00000040018	Cox15	0.00253556	3.95626
ENSMUSG00000066721	Zfp575	0.00251754	3.95844
ENSMUSG00000061718	Ppp1r1b	0.0025165	3.95901
ENSMUSG00000032786	Alas1	0.00251208	3.95991
ENSMUSG00000058207	Serpina3k	0.0024443	3.96739
ENSMUSG00000074491	Clec4g	0.00244179	3.96812
ENSMUSG00000029556	Hnf1a	0.00237363	3.97582
ENSMUSG00000031444	F10	0.00235095	3.97858
ENSMUSG00000031765	Mt1	0.00232868	3.98133
ENSMUSG00000018387	Shroom1	0.00232368	3.98232
ENSMUSG00000033581	Igf2bp2	0.00225395	3.99004
ENSMUSG00000078652	Psme3	0.00225395	3.99051
ENSMUSG00000028214	Gem	0.00223037	3.99359
ENSMUSG00000020697	Lig3	0.00214726	4.00394
ENSMUSG00000028367	Txn1	0.00214726	4.00436
ENSMUSG00000062760	1810041L15Rik	0.00205043	4.01581
ENSMUSG00000075543	Urad	0.00203327	4.01828
ENSMUSG00000058809	Gm12141	0.00201866	4.02047
ENSMUSG00000041660	Bbox1	0.00198374	4.02556
ENSMUSG00000015083	C8g	0.00198158	4.02638
ENSMUSG00000085972	1110028F11Rik	0.00196351	4.02944
ENSMUSG00000061959	Ces1e	0.00195986	4.03037
ENSMUSG00000063171	Rps4y2	0.00195583	4.03142
ENSMUSG00000005251	Ripk4	0.0019557	4.03236
ENSMUSG00000026496	Parp1	0.00194437	4.03422
ENSMUSG00000035769	Xylb	0.00190791	4.03916
ENSMUSG00000051748	1100001G20Rik	0.00190791	4.03944
ENSMUSG00000037095	Lrg1	0.00190614	4.04038
ENSMUSG00000032028	Nxpe2	0.00187628	4.04458
ENSMUSG00000038745	Nlrp6	0.00187628	4.04494
ENSMUSG00000036492	Rnf39	0.00185662	4.0481
ENSMUSG00000079662	Ntn3	0.00185083	4.05029
ENSMUSG00000060317	Acnat2	0.00184204	4.05191
ENSMUSG00000021336	Slc17a4	0.00184204	4.05226
ENSMUSG00000006567	Atp7b	0.00175853	4.06376
ENSMUSG00000060572	Mfap2	0.00172288	4.06971
ENSMUSG00000021335	Slc17a1	0.00171663	4.07142
ENSMUSG00000031383	Dusp9	0.00170013	4.07418
ENSMUSG00000032561	Acpp	0.00165193	4.08138

ENSMUSG00000027220	Syt13	0.00157686	4.09422
ENSMUSG00000005968	Tuft1	0.00155354	4.09974
ENSMUSG00000001435	Col18a1	0.00154724	4.1012
ENSMUSG00000023832	Acat2	0.00154127	4.10262
ENSMUSG00000099146	RP23-138F21.1	0.00152473	4.10563
ENSMUSG00000056035	Cyp3a11	0.00151994	4.10688
ENSMUSG00000047638	Nr1h4	0.00150996	4.10945
ENSMUSG00000028664	Ephb2	0.00144466	4.1207
ENSMUSG00000030968	Pdilt	0.00143706	4.12244
ENSMUSG00000045136	Tubb2b	0.00142667	4.12494
ENSMUSG00000060314	Zfp941	0.00142039	4.12777
ENSMUSG00000045314	Sowahb	0.00140005	4.13162
ENSMUSG00000081603	Gm14681	0.00139645	4.13275
ENSMUSG00000031781	Ciapin1	0.00138779	4.13471
ENSMUSG00000055312	0610012H03Rik	0.00137798	4.13688
ENSMUSG00000063919	Srrm4	0.00137732	4.13752
ENSMUSG00000048485	Zbtb8b	0.00136351	4.14037
ENSMUSG00000074604	Mgst2	0.00136351	4.14091
ENSMUSG00000001665	Gstt3	0.00136351	4.1411
ENSMUSG00000045733	Sprn	0.00136351	4.14126
ENSMUSG00000032554	Trf	0.0013393	4.14664
ENSMUSG00000037025	Foxa2	0.0013393	4.14713
ENSMUSG00000073565	Prr16	0.00130923	4.15293
ENSMUSG00000058216	BC021614	0.00127321	4.15985
ENSMUSG00000050541	Adra1b	0.00127193	4.16063
ENSMUSG00000031283	Chrdl1	0.00127039	4.16145
ENSMUSG00000031976	Urb2	0.00126951	4.16216
ENSMUSG00000091553	Serpina3e-ps	0.00126686	4.16318
ENSMUSG00000019368	Sec14l4	0.00126156	4.16469
ENSMUSG00000035385	Ccl2	0.00126066	4.16541
ENSMUSG00000028185	Dnase2b	0.00123943	4.17038
ENSMUSG00000061292	Cyp3a59	0.00123943	4.17082
ENSMUSG00000001155	Ftcd	0.00118717	4.18241
ENSMUSG00000032291	Crabp1	0.00118037	4.18428
ENSMUSG00000097203	4732419C18Rik	0.00115586	4.19016
ENSMUSG00000026471	Mr1	0.00115586	4.19062
ENSMUSG00000066979	Bub3	0.00115586	4.19073
ENSMUSG00000040536	Necab1	0.00115505	4.19201
ENSMUSG00000025279	Dnase1l3	0.00115111	4.19335
ENSMUSG00000067225	Cyp2c54	0.00114148	4.19583
ENSMUSG00000028655	Mfsd2a	0.00111906	4.20146
ENSMUSG00000037440	Vnn1	0.0010845	4.20912
ENSMUSG00000026245	Farsb	0.00107362	4.21197
ENSMUSG00000022304	Dpys	0.00107362	4.21246
ENSMUSG00000052534	Pbx1	0.00105506	4.21705
ENSMUSG00000066363	Serpina3f	0.00105225	4.21823
ENSMUSG00000027016	Zfp385b	0.00102359	4.22579
ENSMUSG00000027996	Sfrp2	0.000997779	4.23309
ENSMUSG00000052131	Akr1b7	0.00098895	4.23567
ENSMUSG00000089876	Tmem102	0.000978948	4.23853
ENSMUSG00000009376	Met	0.000976897	4.23959
ENSMUSG00000028217	Cdh17	0.000973526	4.24095
ENSMUSG00000044339	Alkbh2	0.000973526	4.24149
ENSMUSG00000073555	Gm4951	0.000938601	4.25208
ENSMUSG00000028980	H6pd	0.000936997	4.25306
ENSMUSG00000020183	Cpm	0.000923881	4.25681
ENSMUSG00000022330	Osr2	0.000923881	4.25715
ENSMUSG00000092021	Gbp11	0.000914026	4.2604
ENSMUSG00000061048	Cdh3	0.000899629	4.26454
ENSMUSG00000030359	Pzp	0.000899629	4.26491
ENSMUSG00000026890	Lhx6	0.000884565	4.26997
ENSMUSG00000020679	Hnf1b	0.000850199	4.27955
ENSMUSG00000020182	Ddc	0.000836641	4.28373
ENSMUSG00000066361	Serpina3c	0.000836641	4.28424
ENSMUSG00000030613	Ccdc90b	0.000835348	4.28528
ENSMUSG00000030638	Sh3gl3	0.000832574	4.28663
ENSMUSG00000037138	Aff3	0.000832574	4.28697
ENSMUSG00000048856	Slc25a47	0.000830884	4.28891
ENSMUSG00000020258	Glyctk	0.000830884	4.28911
ENSMUSG00000022790	Igsf11	0.000822016	4.29253
ENSMUSG00000078817	Nlrp12	0.000804646	4.29788
ENSMUSG00000027068	Dhrs9	0.000790502	4.30243
ENSMUSG00000040938	Slc16a11	0.000783178	4.30511

ENSMUSG00000082121	Gm8199	0.000779826	4.30668
ENSMUSG00000033107	Rnf125	0.000777991	4.30845
ENSMUSG00000067813	Xkr9	0.000766155	4.31246
ENSMUSG00000010175	Prox1	0.00075632	4.31595
ENSMUSG00000035305	Ror1	0.000746901	4.31934
ENSMUSG00000069806	Cacng7	0.000746901	4.32037
ENSMUSG00000026272	Agxt	0.000713812	4.33249
ENSMUSG00000026558	Uck2	0.000713344	4.33327
ENSMUSG00000062661	Ncs1	0.000703595	4.33758
ENSMUSG00000040471	Ggt6	0.000698541	4.3398
ENSMUSG00000020534	Shmt1	0.000677084	4.34794
ENSMUSG00000031844	Hsd17b2	0.000674726	4.34935
ENSMUSG00000030551	Nr2f2	0.000674726	4.34956
ENSMUSG00000048087	Gm4737	0.000646728	4.36012
ENSMUSG00000055730	Ces2a	0.000620977	4.37077
ENSMUSG00000023057	Fabp2	0.000595061	4.38269
ENSMUSG00000089678	Agxt2	0.000586616	4.38646
ENSMUSG00000090555	Gm8893	0.000580637	4.38936
ENSMUSG00000028755	Cda	0.000562839	4.39678
ENSMUSG00000039252	Lgi2	0.000562839	4.39679
ENSMUSG00000026980	Ly75	0.000531407	4.41191
ENSMUSG00000060459	Kng2	0.000529757	4.41326
ENSMUSG00000019775	Rgs17	0.000528664	4.41438
ENSMUSG00000055733	Nap1l3	0.00052597	4.41616
ENSMUSG00000034584	Exph5	0.000525945	4.41684
ENSMUSG00000005836	Gata6	0.000521707	4.41995
ENSMUSG00000019312	Grb7	0.000521707	4.42031
ENSMUSG00000010080	Epn3	0.000521707	4.42214
ENSMUSG00000048388	Fam171b	0.000521707	4.4231
ENSMUSG00000057037	Cfhr1	0.000521707	4.42353
ENSMUSG00000079197	Psme2	0.000521707	4.42534
ENSMUSG00000021999	Cpb2	0.000520801	4.42655
ENSMUSG00000000301	Pemt	0.00052016	4.42751
ENSMUSG00000036110	Slc17a2	0.000487477	4.4422
ENSMUSG00000032459	Mrps22	0.000467115	4.45277
ENSMUSG00000037580	Gch1	0.000467115	4.45296
ENSMUSG00000030762	Aqp8	0.000467115	4.45342
ENSMUSG00000034171	Faah	0.000463108	4.45675
ENSMUSG00000051065	Mb21d2	0.000463108	4.45699
ENSMUSG00000030909	Anks4b	0.000463108	4.45815
ENSMUSG00000056758	Hmga2	0.000461285	4.45974
ENSMUSG00000001670	Tat	0.000458495	4.46176
ENSMUSG00000026870	Cutal	0.000458495	4.46196
ENSMUSG00000026822	Lcn2	0.000458495	4.46255
ENSMUSG00000053219	Raet1e	0.000448954	4.46917
ENSMUSG00000022306	Zfpm2	0.000448954	4.46935
ENSMUSG00000054196	Cthrc1	0.000448954	4.46979
ENSMUSG00000022103	Gfra2	0.000441188	4.47534
ENSMUSG00000022219	Cideb	0.000440592	4.47687
ENSMUSG00000051817	Sox12	0.000438137	4.4788
ENSMUSG00000032463	Faim	0.000430161	4.48421
ENSMUSG00000031725	Ces1f	0.000409564	4.49542
ENSMUSG00000017453	Pipox	0.000405981	4.49803
ENSMUSG00000018446	C1qbp	0.000400451	4.5017
ENSMUSG00000052520	Cyp2j5	0.000399861	4.50277
ENSMUSG00000023829	Slc22a1	0.000399861	4.50282
ENSMUSG00000029370	Rassf6	0.000399861	4.50327
ENSMUSG00000047109	Cldn14	0.000399317	4.50534
ENSMUSG00000035451	Foxa1	0.000390345	4.51169
ENSMUSG00000059908	Mug1	0.000389882	4.51272
ENSMUSG00000038366	Lasp1	0.000383779	4.51761
ENSMUSG00000074768	Bhmt	0.000378362	4.52217
ENSMUSG00000050440	Hamp	0.000365051	4.53131
ENSMUSG00000030498	Gas2	0.00035381	4.53949
ENSMUSG00000021273	Fdft1	0.000342881	4.54689
ENSMUSG00000069922	Ces3a	0.00033887	4.55016
ENSMUSG00000021957	Tkt	0.000315579	4.56671
ENSMUSG00000032068	1600029D21Rik	0.000305178	4.57453
ENSMUSG00000006522	Itih3	0.0003041	4.57607
ENSMUSG00000030827	Fgf21	0.000303176	4.57751
ENSMUSG00000036218	Pdzrn4	0.000294359	4.58449
ENSMUSG00000078650	G6pc	0.000290831	4.58781
ENSMUSG00000027048	Abcb11	0.000285947	4.59297

ENSMUSG00000036412	Arsi	0.000285947	4.59363
ENSMUSG00000018166	Erbb3	0.000285947	4.59386
ENSMUSG00000057933	Gsta2	0.000276922	4.60211
ENSMUSG00000021747	4930452B06Rik	0.000276922	4.60231
ENSMUSG00000032372	Plscr2	0.000272524	4.6071
ENSMUSG00000037942	Crp	0.000270077	4.6098
ENSMUSG00000013415	Igf2bp1	0.000269929	4.61075
ENSMUSG00000019945	1700040L02Rik	0.000258288	4.62074
ENSMUSG00000023046	Igfbp6	0.000257732	4.62202
ENSMUSG00000041449	Serpina3h	0.000257732	4.62329
ENSMUSG00000044206	Vsig4	0.000256593	4.62548
ENSMUSG00000014542	Clec4f	0.000252405	4.62974
ENSMUSG00000049152	Ugt3a2	0.000247856	4.63435
ENSMUSG00000020684	Rasl10b	0.000243258	4.63908
ENSMUSG00000050587	Lrrc4c	0.000242454	4.64063
ENSMUSG00000020102	Slc16a7	0.000238975	4.64447
ENSMUSG00000039653	Baat	0.000237975	4.6462
ENSMUSG00000054422	Fabp1	0.000237975	4.64631
ENSMUSG00000047728	BC025446	0.000237975	4.64709
ENSMUSG00000032564	Cpne4	0.000233386	4.65372
ENSMUSG00000030087	Klf15	0.000232445	4.65543
ENSMUSG00000046959	Slc26a1	0.0002286	4.65975
ENSMUSG00000032591	Mst1	0.0002286	4.65976
ENSMUSG00000022821	Hgd	0.0002286	4.6608
ENSMUSG00000031762	Mt2	0.000222378	4.66811
ENSMUSG00000027761	Aadac	0.000218641	4.67339
ENSMUSG00000084983	Gm11789	0.000218121	4.67478
ENSMUSG00000045659	Plekha7	0.000218121	4.67562
ENSMUSG00000022445	Cyp2d26	0.000217263	4.67741
ENSMUSG00000026295	Spp2	0.000209847	4.68545
ENSMUSG00000085214	0610005C13Rik	0.000205138	4.69194
ENSMUSG00000031271	Serpina7	0.000205138	4.69223
ENSMUSG00000019851	Perp	0.000205138	4.6924
ENSMUSG00000002588	Pon1	0.000205138	4.69282
ENSMUSG00000033688	1300017J02Rik	0.000205138	4.69297
ENSMUSG00000017493	Igfbp4	0.000205138	4.69539
ENSMUSG00000074071	Fam169b	0.000205138	4.69742
ENSMUSG00000033860	Fgg	0.000205138	4.6976
ENSMUSG00000029630	Cyp3a25	0.000205138	4.69859
ENSMUSG00000036334	Igsf10	0.000201654	4.70393
ENSMUSG00000024039	Cbs	0.000200937	4.70562
ENSMUSG00000024331	Dsc2	0.000196135	4.71319
ENSMUSG00000038563	Eftud1	0.00019435	4.71632
ENSMUSG00000029369	Afm	0.000194023	4.71764
ENSMUSG00000028654	Mycl	0.000192337	4.72041
ENSMUSG00000016458	Wt1	0.000183241	4.73125
ENSMUSG00000031298	Gpr64	0.000178208	4.73789
ENSMUSG00000042515	Mum1l1	0.000174964	4.74262
ENSMUSG00000021135	Slc10a1	0.000160025	4.76267
ENSMUSG00000006529	Itih1	0.000159374	4.7645
ENSMUSG00000027359	Slc27a2	0.000159374	4.76481
ENSMUSG00000054889	Dsp	0.000156463	4.77127
ENSMUSG00000020429	Igfbp1	0.000156463	4.77172
ENSMUSG00000066366	Serpina1a	0.000154822	4.7765
ENSMUSG00000062991	Nrg1	0.000154615	4.77782
ENSMUSG00000025105	Bnc1	0.000149068	4.78726
ENSMUSG00000082361	Btc	0.000149068	4.78814
ENSMUSG00000019762	Iyd	0.000145965	4.7938
ENSMUSG00000047228	BC048546	0.000139929	4.80529
ENSMUSG00000025207	Sema4g	0.000139929	4.80564
ENSMUSG00000035186	Ubd	0.000139627	4.80789
ENSMUSG00000081640	Rplp0-ps1	0.000138191	4.81216
ENSMUSG00000030884	Uqcrc2	0.000123287	4.83712
ENSMUSG00000056665	Them6	0.000120632	4.84368
ENSMUSG00000057113	Npm1	0.000120481	4.84505
ENSMUSG00000063696	Gm8730	0.000120481	4.84542
ENSMUSG00000038812	Trmt112	0.000120481	4.84643
ENSMUSG00000022766	Serpind1	0.000117993	4.85307
ENSMUSG00000063354	Slc39a4	0.000115978	4.85832
ENSMUSG00000032179	Bmp5	0.000109313	4.87235
ENSMUSG00000024863	Mbl2	0.000104651	4.88212
ENSMUSG00000031138	F9	9.43915E-05	4.90359
ENSMUSG00000005268	Prlr	9.06355E-05	4.91274

ENSMUSG00000021922	Itih4	8.91703E-05	4.91831
ENSMUSG00000028356	Ambp	0.0000871	4.9253
ENSMUSG00000074336	Apoc4	8.68245E-05	4.92713
ENSMUSG00000040413	Timd2	8.58034E-05	4.93066
ENSMUSG00000025481	Urah	8.46982E-05	4.93442
ENSMUSG00000030738	Eif3c	8.45153E-05	4.93607
ENSMUSG00000031722	Hp	8.34889E-05	4.93969
ENSMUSG00000021539	Lect2	7.93472E-05	4.95336
ENSMUSG00000070858	Gm1673	7.78106E-05	4.9597
ENSMUSG00000038224	Serpinf2	7.72694E-05	4.96234
ENSMUSG00000024164	C3	7.53839E-05	4.96842
ENSMUSG00000029032	Arhgef16	7.53839E-05	4.96924
ENSMUSG00000030895	Hpx	0.000075293	4.97126
ENSMUSG00000028979	Masp2	7.39745E-05	4.976
ENSMUSG00000047230	Cldn2	7.13004E-05	4.98444
ENSMUSG00000022464	Slc38a4	7.07931E-05	4.98715
ENSMUSG00000009646	Pla2g12b	7.02064E-05	4.9901
ENSMUSG00000027513	Pck1	6.84006E-05	4.99647
ENSMUSG00000030972	Acsm5	6.84006E-05	4.99664
ENSMUSG00000051716	Apon	6.80389E-05	5.00021
ENSMUSG00000025991	Cps1	6.69904E-05	5.00596
ENSMUSG00000031445	Proz	6.69904E-05	5.00612
ENSMUSG00000057751	Megf6	6.69904E-05	5.00739
ENSMUSG00000027249	F2	6.59089E-05	5.01329
ENSMUSG00000061947	Serpina10	6.59089E-05	5.01388
ENSMUSG00000041237	Pklr	6.59089E-05	5.01594
ENSMUSG00000060407	Cyp2a12	0.00006544	5.01895
ENSMUSG00000052396	Mogat2	6.38823E-05	5.02502
ENSMUSG00000037798	Mat1a	6.23256E-05	5.03121
ENSMUSG00000046352	Gjb2	6.15819E-05	5.03498
ENSMUSG00000064225	Paqr9	6.14965E-05	5.03673
ENSMUSG00000017344	Vtn	6.14965E-05	5.03797
ENSMUSG00000000120	Ngfr	6.14965E-05	5.03809
ENSMUSG00000030131	Mug2	5.85932E-05	5.05048
ENSMUSG00000029380	Cxcl1	5.71419E-05	5.05679
ENSMUSG00000026715	Serpinc1	5.66766E-05	5.05988
ENSMUSG00000044254	Pcsk9	5.66766E-05	5.06103
ENSMUSG00000031775	Pllp	5.66766E-05	5.06235
ENSMUSG00000067235	H2-Q10	5.63967E-05	5.06549
ENSMUSG00000030980	Knop1	5.63967E-05	5.0668
ENSMUSG00000053414	Hunk	5.52359E-05	5.07262
ENSMUSG00000020467	Efemp1	0.000054371	5.07723
ENSMUSG00000031594	Fgl1	5.34712E-05	5.08201
ENSMUSG00000033831	Fgb	5.34712E-05	5.08296
ENSMUSG00000068876	Cgn	5.24143E-05	5.08907
ENSMUSG00000041540	Sox5	5.23207E-05	5.09107
ENSMUSG00000029377	Ereg	5.23207E-05	5.09255
ENSMUSG00000085442	Gm3362	5.23207E-05	5.09322
ENSMUSG00000026866	Kynu	5.14719E-05	5.09922
ENSMUSG00000025127	Gcgr	5.14719E-05	5.10047
ENSMUSG00000074828	Gm10768	5.10198E-05	5.10607
ENSMUSG00000061808	Ttr	4.96646E-05	5.11292
ENSMUSG00000019866	Aim1	4.95849E-05	5.11499
ENSMUSG00000021091	Serpina3n	4.95849E-05	5.11612
ENSMUSG00000020098	Pcbd1	4.88349E-05	5.12328
ENSMUSG00000043789	Vwce	4.84401E-05	5.12851
ENSMUSG00000026576	Atp1b1	4.82389E-05	5.13116
ENSMUSG00000025497	Cdhr5	4.77867E-05	5.13482
ENSMUSG00000022875	Kng1	4.77867E-05	5.13535
ENSMUSG00000033715	Akr1c14	4.77867E-05	5.13699
ENSMUSG00000060961	Slc4a4	4.77867E-05	5.14002
ENSMUSG00000026874	Hc	4.77867E-05	5.14072
ENSMUSG00000079563	Pglyrp2	4.56952E-05	5.15496
ENSMUSG00000000049	Apoh	4.56952E-05	5.15569
ENSMUSG00000079012	Serpina3m	4.56952E-05	5.15718
ENSMUSG00000058022	Adtrp	4.44251E-05	5.16638
ENSMUSG00000057400	Ces1c	4.37731E-05	5.17264
ENSMUSG00000024479	Mal2	4.37731E-05	5.17318
ENSMUSG00000021210	Akr1c6	4.37731E-05	5.17411
ENSMUSG00000059456	Ptk2b	4.37731E-05	5.17661
ENSMUSG00000028871	Rspo1	4.25218E-05	5.18743
ENSMUSG00000024827	Gldc	4.14014E-05	5.19688
ENSMUSG00000028001	Fga	3.91537E-05	5.20953

ENSMUSG00000026051	1500015O10Rik	3.91537E-05	5.21171
ENSMUSG00000021751	Acox2	3.91537E-05	5.21411
ENSMUSG00000057074	Ces1g	3.71134E-05	5.22644
ENSMUSG00000023243	Kcnk5	3.71134E-05	5.227
ENSMUSG00000026405	C4bp	3.68617E-05	5.23742
ENSMUSG00000024987	Cyp26a1	3.68243E-05	5.24012
ENSMUSG00000005547	Cyp2a5	3.68243E-05	5.24461
ENSMUSG00000059481	Plg	3.60827E-05	5.25161
ENSMUSG00000029368	Alb	3.60773E-05	5.25429
ENSMUSG00000017950	Hnf4a	3.49689E-05	5.26271
ENSMUSG00000029695	Aass	0.000034877	5.26592
ENSMUSG00000020609	Apob	3.45385E-05	5.27047
ENSMUSG00000029859	Epha1	3.22774E-05	5.28568
ENSMUSG00000040564	Apoc1	2.67195E-05	5.32709
ENSMUSG00000027173	Depdc7	2.46132E-05	5.34659
ENSMUSG00000048826	Dact2	2.44958E-05	5.35045
ENSMUSG00000022868	Ahsg	2.26089E-05	5.36797
ENSMUSG00000034926	Dhcr24	2.23244E-05	5.37334
ENSMUSG00000000247	Lhx2	2.17627E-05	5.38106
ENSMUSG00000047797	Gjb1	2.17627E-05	5.38142
ENSMUSG00000023176	Cpn2	2.17627E-05	5.38967
ENSMUSG00000035472	Slc25a21	2.17627E-05	5.39338
ENSMUSG00000054932	Afp	2.17627E-05	5.39351
ENSMUSG00000020884	Asgr1	2.17627E-05	5.39774
ENSMUSG00000073988	Ttpa	2.17627E-05	5.40066
ENSMUSG00000034450	Gulo	2.15654E-05	5.40984
ENSMUSG00000027690	Slc2a2	2.15654E-05	5.41002
ENSMUSG00000044641	Pard6b	2.14563E-05	5.41821
ENSMUSG00000058952	Cfi	2.14563E-05	5.41982
ENSMUSG00000030088	Aldh1l1	2.11687E-05	5.43174
ENSMUSG00000030772	Dkk3	2.02755E-05	5.44418
ENSMUSG00000021492	F12	1.43403E-05	5.50966
ENSMUSG00000028179	Cth	1.43403E-05	5.51147
ENSMUSG00000076441	Ass1	1.39761E-05	5.52276
ENSMUSG00000046687	Gm5424	1.04936E-05	5.57729
ENSMUSG00000037166	Ppp1r14a	0.000010469	5.58221
ENSMUSG00000027832	Ptx3	8.83107E-06	5.61633
ENSMUSG00000024990	Rbp4	8.3521E-06	5.63069
ENSMUSG00000000385	Tmprss2	8.0389E-06	5.64213
ENSMUSG00000032475	Nck1	8.0389E-06	5.6447
ENSMUSG00000015854	Cd5l	6.01551E-06	5.70187
ENSMUSG00000029371	Cxcl5	5.41526E-06	5.72498
ENSMUSG00000032348	Gsta4	5.2695E-06	5.74629
ENSMUSG00000067219	Nipal1	4.15965E-06	5.79817
ENSMUSG00000084128	Esrp2	4.15965E-06	5.80098
ENSMUSG00000029727	Cyp3a13	2.69248E-06	5.88344
ENSMUSG00000026579	F5	2.39534E-06	5.90949
ENSMUSG00000024292	Cyp4f14	2.02747E-06	5.94387
ENSMUSG00000005089	Slc1a2	2.02639E-06	5.95885
ENSMUSG00000031488	Rab11fip1	1.55026E-06	6.03668
ENSMUSG00000022756	Slc7a4	1.4386E-06	6.05849
ENSMUSG00000016194	Hsd11b1	1.4386E-06	6.06807
ENSMUSG00000042377	Fam83g	5.31554E-07	6.23764
ENSMUSG00000091345	Col6a5	1.51706E-07	6.45437
ENSMUSG00000066357	Wdr6	1.23396E-07	6.49868
ENSMUSG00000021097	Clmn	1.89013E-08	6.78911
ENSMUSG00000026069	Il1rl1	1.89013E-08	6.79437
ENSMUSG00000027239	Mdk	6.10883E-09	7.00038
ENSMUSG00000023043	Krt18	4.38519E-09	7.06811
ENSMUSG00000039323	Igfbp2	4.41475E-10	7.47187
