# Supplementary material for: CD44 Contributes to the Regulation of MDR1 Protein and Doxorubicin Chemoresistance in Osteosarcoma
Source: Int J Mol Sci. 2022 Aug 3;23(15):8616. doi: 10.3390/ijms23158616 (PMC9368984; doi:10.3390/ijms23158616)
Supplement: Supplementary file 1 [file ijms-23-08616-s001.zip › Table S2.pdf]

Table S2. List of molecular pathways implicated in the regulation of MDR1 by CD44. The list was generated based on PubMed search using CD44 and MDR1 as key words. State on December 2021. CD44-HA delineates CD44 – hyaluronic acid (HA) interaction.

Molecular pathway	Cells	Reference
CD44 – inhibition of FBXO21-mediated ubiquitination and proteasomal degradation of MDR1	human breast tumor cells (MCF-7) and human ovarian carcinoma cell line (SKA)	[57]
Formation of CD44-ankyrin-MDR1 complex – Mdr1 activation	human breast tumor cells (MCF-7) and human ovarian tumor cells (SK-OV-3.ipl)	[12]
CD44-HA – up-regulation and activation of p300 (a histone acetyltransferase) - acetylation of β-catenin - activation of β-catenin-associated T-cell factor/lymphocyte enhancer factor (TCF/LEF) transcriptional co-activation - Mdr1 gene expression	human breast tumor cells (MCF-7)	[13]
CD44-HA - ErbB2 complex activation - phosphoinositide 3-kinase activity – Mdr1 gene expression	multidrug-resistant MCF-7/Adr human breast carcinoma cells	[11]
CD44-HA – Nanog/Stat3 transcriptional activation – Mdr1 gene expression	human breast tumor cells (MCF-7) and human ovarian tumor cells (SK-OV-3.ipl)	[12]
CD44-HA – (PKCepsilon activation)- Nanog/Stat-3 transcriptional activation - microRNA-21- down-regulation of PDCD4- MDR1 gene expression with concomitant upregulation of inhibitors of the apoptosis family of proteins (IAPs)	breast tumor cell line MCF-7 head and neck squamous cell carcinoma (HNSCC)	[14,56]
